# Photoactivatable Agonist–Antagonist Pair as a Tool for Precise Spatiotemporal Control of Serotonin Receptor 2C Signaling

**DOI:** 10.1021/acschemneuro.3c00290

**Published:** 2023-09-18

**Authors:** Spencer T. Kim, Emma J. Doukmak, Michelle Shanguhyia, Dylan J. Gray, Rachel C. Steinhardt

**Affiliations:** Syracuse University, Syracuse, New York 13244, United States

**Keywords:** photocaging, serotonin, GPCR, synapse, calcium signaling, 5-HT_2C_

## Abstract

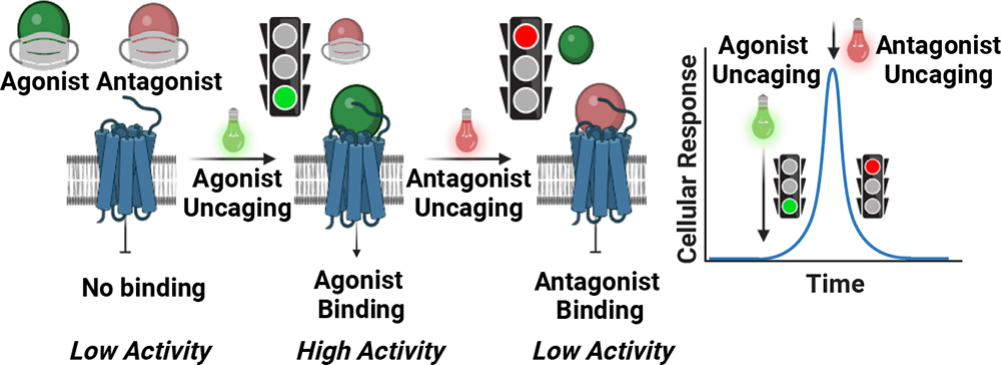

Orthogonal recreation of the signaling profile of a chemical synapse is a current challenge in neuroscience. This is due in part to the kinetics of synaptic signaling, where neurotransmitters are rapidly released and quickly cleared by active reuptake machinery. One strategy to produce a rapid rise in an orthogonally controlled signal is via photocaged compounds. In this work, photocaged compounds are employed to recreate both the rapid rise and equally rapid fall in activation at a chemical synapse. Specifically, a complementary pair of photocages based on BODIPY were conjugated to a 5-HT_2C_ subtype-selective agonist, WAY-161503, and antagonist, *N*-desmethylclozapine, to generate “caged” versions of these drugs. These conjugates release the bioactive drug upon illumination with green light (agonist) or red light (antagonist). We report on the synthesis, characterization, and bioactivity testing of the conjugates against the 5-HT_2C_ receptor. We then characterize the kinetics of photolysis quantitatively using HPLC and qualitatively in cell culture conditions stimulating live cells. The compounds are shown to be stable in the dark for 48 h at room temperature, yet photolyze rapidly when irradiated with visible light. In live cells expressing the 5-HT_2C_ receptor, precise spatiotemporal control of the degree and length of calcium signaling is demonstrated. By loading both compounds in tandem and leveraging spectral multiplexing as a noninvasive method to control local small-molecule drug availability, we can reproducibly initiate and suppress intracellular calcium flux on a timescale not possible by traditional methods of drug dosing. These tools enable a greater spatiotemporal control of 5-HT_2C_ modulation and will allow for more detailed studies of the receptors’ signaling, interactions with other proteins, and native physiology.

## Introduction

Serotonergic signaling is essential for processes ranging from homeostasis to executive function and emotional regulation. In mammals, serotonin is thought to play a modulatory role in almost every physiological function.^1^ Dysfunction in the serotonergic signaling system is associated with anxiety, depression, schizophrenia, migraines, autism, Parkinson’s disease, and Alzheimer’s disease.^2−8^ Due to its importance, the serotonergic system is the target of many pharmaceuticals, such as the antidepressant drug families of monoamine oxidase inhibitors, selective serotonin reuptake inhibitors, and tricyclic antidepressants.^9,10^ There are seven types of serotonin receptors and a number of subtypes. In dysfunction, these diverse receptors contribute to many diseases and therefore provide important therapeutic potential for drugs targeted to individual serotonin receptor subtypes.^11^ Each subtype also contributes to varied physiological states; for example, subtype-selective drugs can treat symptoms of migraine, anxiety, psychosis, or in contrast, can cause hallucinations.^11^ In fundamental research, studies are underway to determine the effect of stimulating a serotonin receptor subtype on brain dynamics and the origin of the brain’s flexibility to produce different states.^12^

The role of serotonergic signaling in health and disease is an area of intense research. However, there is a lack of tools to enable researchers to tease apart the signaling processes in the exquisitely interconnected networks controlled by the serotonin receptor family. One such challenge is that of recreating the unique kinetics of the chemical synapse, where a bolus of neurotransmitter is rapidly released and then quickly cleared by active reuptake machinery. Mimicking this system requires a tool that can provide an incredibly rapid rise in signal followed by an equally rapid fall. To understand how signals from distinct members of the serotonin receptor family affect physiological response, such a tool should ideally be selective for a single subtype.

One strategy to produce a rapid rise in an orthogonally controlled signal is via photocaged compounds.^13^ Photocages are photoremovable protecting groups that greatly reduce a compound’s affinity for its receptor. Removal of this protecting group with the appropriate wavelength and intensity of light can result in the delivery of the active compound in less than a microsecond.^14^ A number of compounds important for neuroscience have been caged, including serotonin (NPEC-5-HT, BHQ-O-5-HT, [Ru(bpy)_2_(PMe_3_)(5-HT)]^2+^), dopamine (BBHCM-DA, CNB-DA, CNV-DA, NPEC-DA, RuBi-DA), and subtype-selective dopamine receptor D2/D3 antagonists dechloroeticlopride and sulpiride.^15−22^ Thus, although dopamine and serotonin have been caged multiple times (including commercial products), there is almost no work disclosing caged subtype-selective modulators.

**Figure 1:**
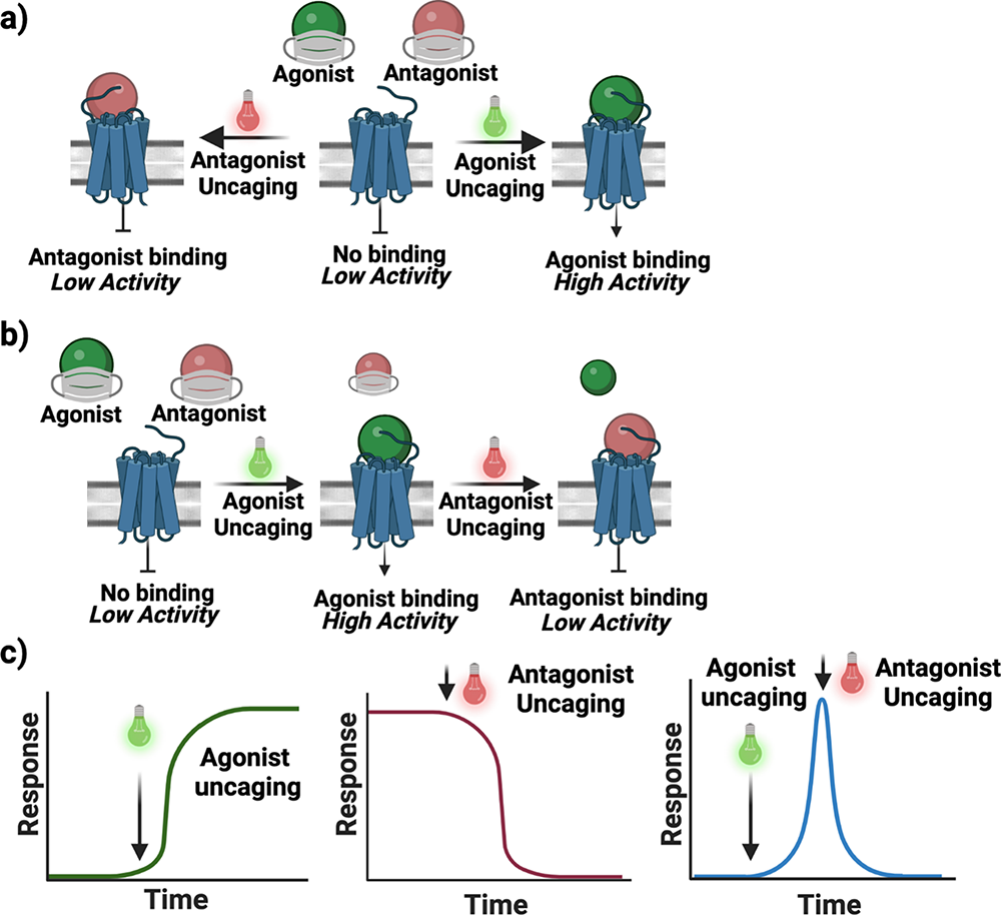
Concept of tight receptor control via photocaged agonist/antagonist pairing. (a) Uncaging of a photocaged agonist or antagonist unveils the hidden biological activity of the drug, rendering its activity controllable by the uncaging wavelengths of light. In one mode, the agonist may be uncaged to initiate receptor signaling. In a second mode, in the presence of free agonist, uncaging the antagonist results in a decrease in receptor activity. (b) Sequential uncaging of agonist and antagonists with suitable potencies results in agonism/antagonism. (c) Using light as a trigger, extremely rapid signal induction and reduction are possible, also with high spatial resolution.

Here, we report on a matched pair of caged subtype-selective serotonin receptor agonist and antagonist for serotonin receptor 5-HT_2C_. The agonist, WAY161503, is caged with a green light-responsive BODIPY photocage, and the antagonist, *N*-desmethylclozapine, is caged with a red light-responsive BODIPY photocage. Illumination with a green laser provides the agonist and red laser unveils the antagonist. This activity is demonstrated in HEK293T cells expressing human 5-HT_2C_ (Figure 1a). This spectral multiplexing also permits us to use both probes in the same experiment, using a green light to deliver an activating bolus of an agonist and a red light to deliver a deactivating bolus of an antagonist (Figure 1b,c). We show that this may be used to target single 5-HT_2C_-transfected HEK293T cells such that we can observe a rise in a stimulation-induced intracellular calcium flux, which may then be rapidly quenched with the uncaging of the antagonist.

## Results and Discussion

### Synthesis of Photocaged Probes

**Scheme 1:**
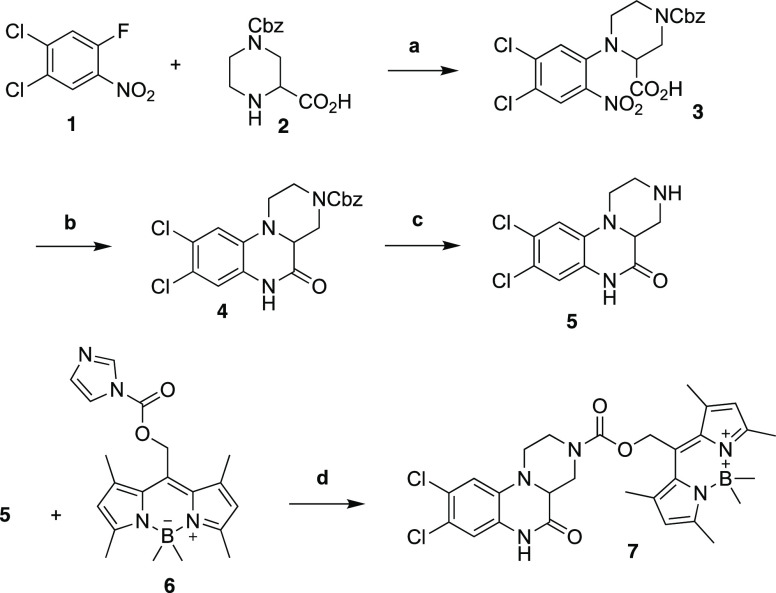
Synthesis of WinterGreen-WAY-161503 (**7**) (a) NEt_3_, DMSO, 50 °C 5 h, 25 °C 16 h, 60%; (b) Fe, AcOH, 60 °C 3 h, 50%; (c) H_2_, Pd/C 1:1 THF:H_2_O, 25 °C 3 h, 66%; (d) NEt_3_, THF 25 °C 24 h, 58%.

We prepared photocaged WAY161563 by first synthesizing the drug and then conjugating it to the photocage. The synthesis of WAY161563 followed the general procedure of Welmaker et al. with modifications (Scheme 1).^23^ Accordingly, a nucleophilic aromatic substitution reaction with 1,2-dichloro-4-fluoro-5-nitrobenzene **1** and commercially available 4-benzyloxycarbonylpiperazine-2-carboxylic acid **2** formed **3** in a 60% yield. Tandem Bechamp reduction and cyclization of **3** formed **4** in 50% yield. The Cbz-amine of **4** was reduced with palladium on carbon under a hydrogen atmosphere to yield racemic **5** (WAY-161503) in 66% yield. As 5-HT_2C_ receptors have been shown not to discriminate between the two enantiomers, they are typically not separated when preparing this drug.^23^ To install the cage, WAY161503 was condensed with commercially available WinterGreen photocage carbonyldiimidazole adduct **6** following the method of Peterson et al. to provide the caged compound **7** in 58% yield.^24−26^

**Scheme 2:**
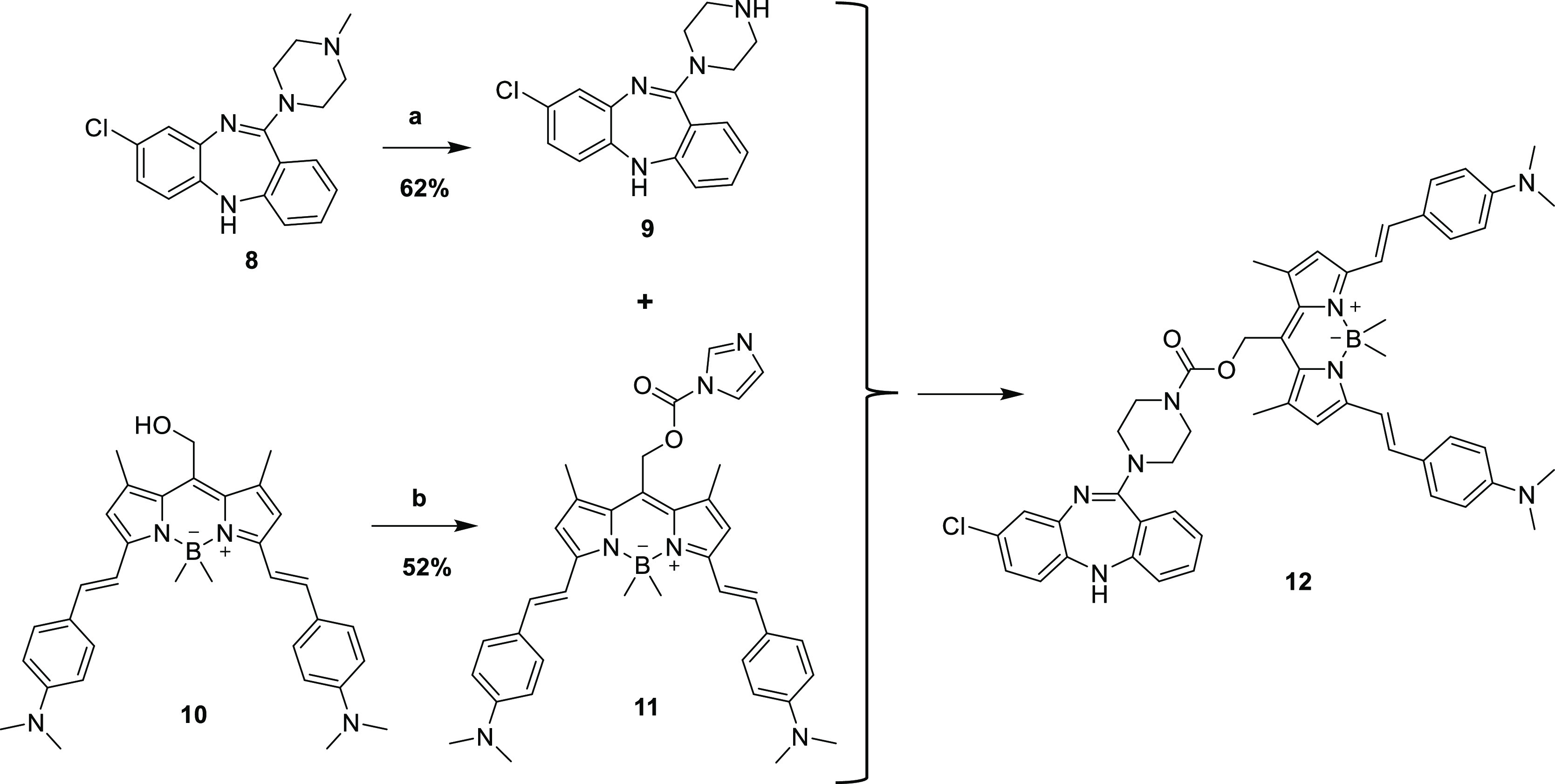
Synthesis of WinterRed-*N*-desmethylclozapine (**12**) (a) 1-Chloroethyl chloroformate, 1,2-dichloroethane reflux 24 h, then MeOH 50 °C 2 h, 62%; (b) CDI, NEt_3_, DIPEA, THF, 25 °C 2 h then **9**, 48 h, 52%.

Caged antagonist *N*-desmethylclozapine was synthesized by first demethylating clozapine following the method of McRobb et al.^27^ Briefly, 1-chloroethyl chloroformate was added to a solution of clozapine **8** and heated to reflux for 24 h. The crude residue was dissolved in methanol and heated at 50 °C for 2 h to yield *N*-desmethylclozapine **9** in 53% yield. Commercially available WinterRed photocage **10** was condensed with carbonyldiimidazole to form the carbamoyl imidazole adduct **11**.^24^ The adduct is then incubated with desmethylclozapine **9** to provide WinterRed photocaged desmethylclozapine **12** in 52% yield (Scheme 2).

### Photochemistry

While there are many known photocages, of particular importance to our synthetic design were amine-compatible photocages due to the necessity of the amine in the active forms of WAY-161563 and *N*-desmethylclozapine. The amine photocages we selected covalently shield the amine with a BODIPY cage, through a cleavable carbamate linker. Upon irradiation, this generates an ion-paired intermediate between the carbocation of the cage and the carbamate anion pendant on the caged drug. Once a solvated ion pair is generated, the carbocation rapidly reacts with water to generate the BODIPY alcohol,^28^ and the carbamate anion on the drug undergoes elimination to the amine^29^ and carbon dioxide. The quantum yield of the release of leaving groups by WinterRed and WinterGreen has been previously determined by ferrioxalate actinometry to be 0.11% and 5.5%, respectively.^24^

**Figure 2:**
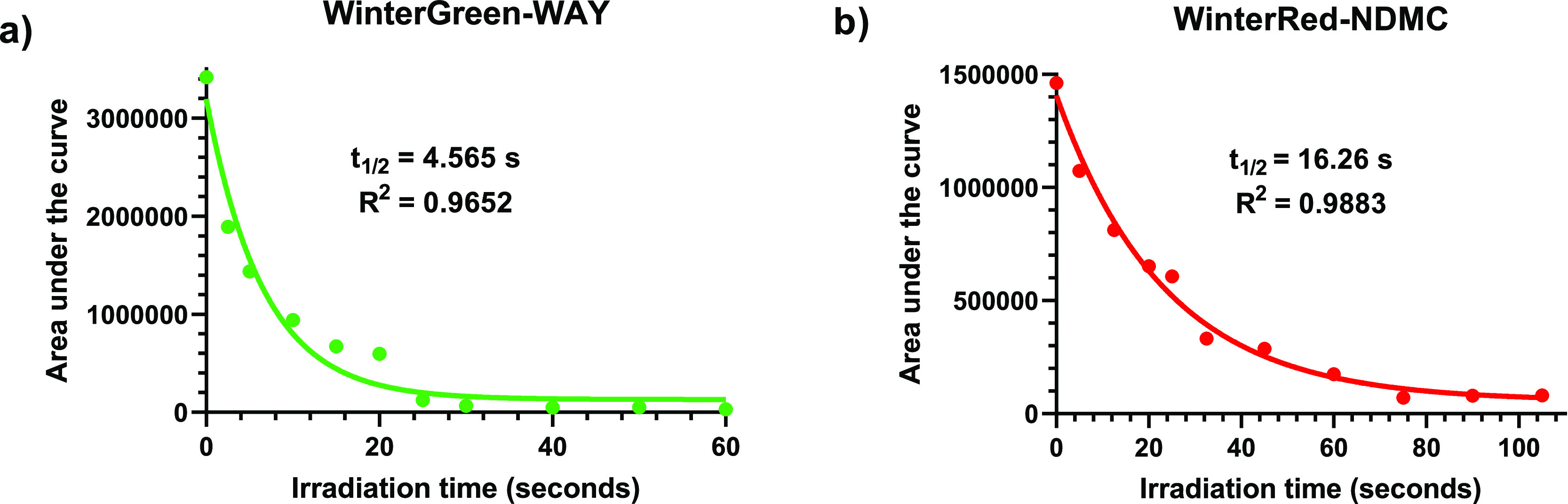
Photolysis kinetics. Uncaging photolysis and subsequent release of payload monitored by HPLC. (a) WinterGreen-WAY-161503 (**5**). (b) WinterRed-NDMC (**12**).

To determine the uncaging rates, we employed reaction monitoring by HPLC, where we detected the disappearance of photocage–pharmacophore conjugate with increasing irradiation time. Fluorescence spectra of each compound in acetonitrile revealed a WinterGreen-WAY excitation maxima of 491 nm and emission maxima at 529 nm. WinterRed-NDMC was far-red shifted with an excitation of 683 nm and emission maxima at 730 nm. Using a mercury-arc lamp as a photolysis light source, WinterGreen-WAY (Figure 2a) photolyzed with a half-life of 4.565 s (first-order exponential decay *R*^2^ = 0.9652) in dimethyl sulfoxide (DMSO). The predominant products of the reaction were BODIPY alcohol and free WAY-161503 with no significant amounts of other photoproducts detected. Yield of uncaging was quantitative at irradiation times greater than 25 s. WinterRed-NDMC (Figure 2b) photolyzed with a half-life of 16.26 s (first-order exponential decay *R*^2^ = 0.9883) in ethyl acetate. No significant photoproducts were detectable upon uncaging. Yield of uncaging was quantitative at irradiation times greater than 75 s. The nearly one order of magnitude increase in half-life is likely explained by the significantly lower quantum yield of WinterRed photocage. The half-life of both compounds was deemed to be relevant on a biological timescale and indicated that, upon irradiation, payloads of WAY-161503 and NDMC could be released at a concentration relevant for 5-HT_2C_ receptor modulation.

To test the susceptibility of each compound toward spontaneous hydrolysis, WinterGreen-WAY and WinterRed-NDMC were stored and protected from light as a 1 mg/mL solution in 60% DMSO in phosphate buffer saline (pH 7.4). The concentration and purity of both solutions remained unchanged after 24 and 48 h in the dark at room temperature, indicated by HPLC. This result, combined with the biologically relevant half-life of each compound, encouraged us to further assay our photocaged compounds for bioactivity at the 5-HT_2C_ receptor.

### Biochemical Characterization of Drug Uncaging

As with all G protein coupled receptors (GPCRs)s, the 5-HT_2C_ receptor detects extracellular effector molecules to activate intracellular responses. An intracellular signaling pathway that is commonly exploited to measure 5-HT_2C_ activation is β-arrestin signaling. Here, we monitored the β-arrestin pathway to determine whether the installed photocages effectively diminished the binding capacity of both pharmacophores beyond any physiologically relevant concentration. We reasoned that if we could confirm the activity by the parent pharmacophores and inactivity of the caged drugs through β-arrestin signaling pathways, the caged drugs did not act as ligands for the 5-HT_2C_ receptor.

#### TANGO Assay

β-Arrestin binding is a pathway linked to G-protein signal transduction.^30^ We measured 5-HT_2C_ activity in response to ligand binding via the PRESTO-TANGO assay, which is based upon an engineered 5-HT_2C_ fusion protein with a cleavable C-terminal transcription factor in HEK293T cells.^31^ Upon ligand binding and recruitment of a protease-tagged β-arrestin, the intracellular C-terminal transcription factor is released, resulting in the transcriptional output of the reporter gene, luciferase, which is quantified.

**Figure 3:**
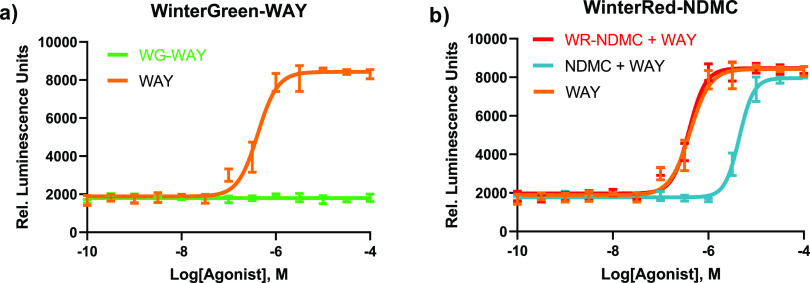
TANGO assay confirmation of loss of bioactivity. Arrestin transcriptional output data from HEK293T cells exogenously expressing human 5-HT_2C_. Luminescence from luciferase expression corresponds to β-arrestin recruitment activity/5-HT_2C_ binding. (a) Wintergreen-WAY-161563 (**5**) vs positive control (uncaged WAY-161563), assayed for 5-HT_2C_ agonism. Green: WinterGreen caged WAY-161563 (**5**); orange: unprotected (free) WAY-161563. (b) WinterRed-NDMC assayed for 5-HT_2C_ antagonism. Red: WinterRed-NDMC (**12**) with WAY-161563 (200 nM constant concentration of WinterRed-NDMC). Teal: uncaged NDMC with WAY-161563 (200 nM constant concentration of NDMC). Orange: unprotected (free) WAY-161563.

The transcriptional output data indicated that at all physiologically relevant concentrations WinterGreen-WAY are inactive for β-arrestin recruitment (Figure 3a). The assay was then performed in antagonist mode by adding a fixed concentration of test compound and observing changes to the curve of the control agonist. Upon incubation with 200 nm WinterRed-NDMC as the test compound, the response curve to WAY-161503 was not shifted, indicating WinterRed-NDMC is inactive as an antagonist for 5-HT_2C_ (Figure 3b, red curve). In contrast, the curve shows a dramatic shift with the addition of uncaged *N-*desmethylclozapine (Figure 3b, teal curve).

### Photoactivation of WinterGreen-WAY and WinterRed-NDMC in Cells Expressing the 5-HT_2C_ Receptor

#### Photoactivation of WinterGreen-WAY (5)

##### Photoactivation in Cell Culture

With positive biochemical and photolysis data in hand, we were encouraged to assay the uncaging efficacy in living cells. Thus, we setup experiments where the culture media was loaded with one or both caged compounds. The single compound experiments track the uncaging of the compounds, while these experiments are designed to control for whether the wavelength and intensity used to uncage one compound also uncages the second (wavelength overlap). First, we needed to confirm that we could monitor levels of intracellular calcium without initiating aberrant photolysis and release of WAY-161503 and NDMC. To do so, HEK293T cells were plated on glass-bottom chambered coverslips and transiently transfected to express both 5-HT_2C_-GFP fusion protein and jRCaMP-1a, a genetically encoded calcium indicator.^32^ Imaging and uncaging photolysis were performed with a laser scanning confocal microscope.

Activation of WinterGreen-WAY or WinterRed-NDMC by laser irradiation occurred at 488 nm or 639 nm, respectively. The 561 nm laser channel was used to observe RCaMP-1a fluorescence before and after irradiation. Media loaded with 3.7 μM WinterGreen-WAY was irradiated at 561 nm for 800 s to check for aberrant release of WAY-161503. No response was observed, so we concluded that WinterGreen-WAY is not activated by extended irradiation of low-intensity 561 nm light. To test for aberrant release of NDMC, media loaded with 2.1 μM WinterRed-NDMC was irradiated at 561 nm for 800 s, then the media was spiked with 3.7 μM WAY-161503. The fluorescence intensity was compared to that of a positive control sample containing no WinterRed-NDMC in the media. No change in fluorescence intensity relative to the control was observed, so we concluded that WinterRed-NDMC is also unaffected by extended irradiation of low intensity 561 nm light.

To test for spectral orthogonality, media containing saturated WinterGreen-WAY was irradiated under the same conditions necessary to uncage WinterRed-NDMC (60% laser power, 639 nm) and observed for aberrant activation. No response was observed, so it was concluded that WinterGreen-WAY was sufficiently spectrally orthogonal to WinterRed-NDMC uncaging conditions. Similarly, media containing saturated WinterRed-NDMC was irradiated under the conditions necessary to uncage WinterGreen-WAY (60% laser power, 488 nm) prior to the addition of 800 nM WAY-161503. The calcium flux curve was compared to a sample containing no WinterRed-NDMC, and no significant change in overall peak fluorescence intensity of shape of calcium flux curve was observed, so it was concluded that WinterRed-NDMC is sufficiently spectrally orthogonal to WinterGreen-WAY uncaging conditions.

##### Kinetics of WinterGreen-WAY (**5**) Activation

**Figure 4:**
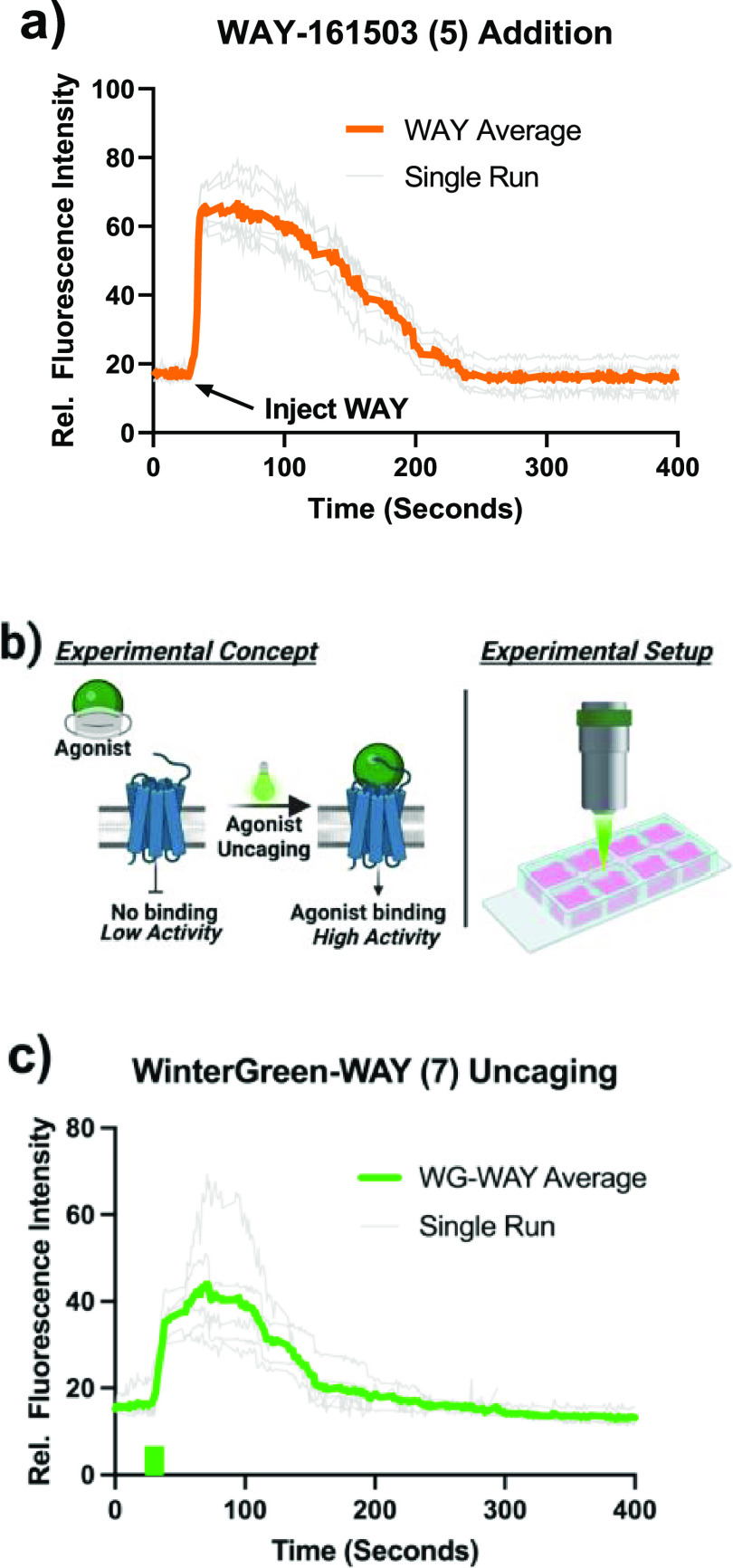
WinterGreen-WAY uncaging. (a) 800 nM WAY-161503 was injected into cell culture media as a control to establish typical calcium flux duration and characteristics under traditional drug dosing conditions. (b) Schematic of experimental design: active agonist is unveiled via a 488 nm laser, which is focused via a confocal microscope into cells plated on a microscope slide. (c) WinterGreen-WAY (**5**) (3.7 μM) uncaging induces a similar calcium mobilization curve and calcium flux duration but with lower maximum fluorescence intensity, indicative of lower local drug concentrations. Green block on the line graph indicates the event of 488 nm irradiation. WG-WAY average curves were generated as the average relative fluorescence intensity of 10 cells where basal fluorescence was normalized.

To study the kinetics of WinterGreen-WAY photolysis under cell culture conditions, calcium response with respect to irradiation time was measured (Figure 4). A region adjacent to a cell of interest was illuminated with a 488 nm laser (the uncaging wavelength), and the subsequent calcium flux due to the binding of uncaged drug to its receptor was monitored via the increase in fluorescence of the calcium indicator protein RCaMP-1a. A 200 ms pulse of high-intensity irradiation was found to be sufficient to elicit calcium signaling (Figure 4b,c). In the presence of 488 nm irradiation but in the absence of WinterGreen-WAY, no calcium response was observed.

**Figure 5:**
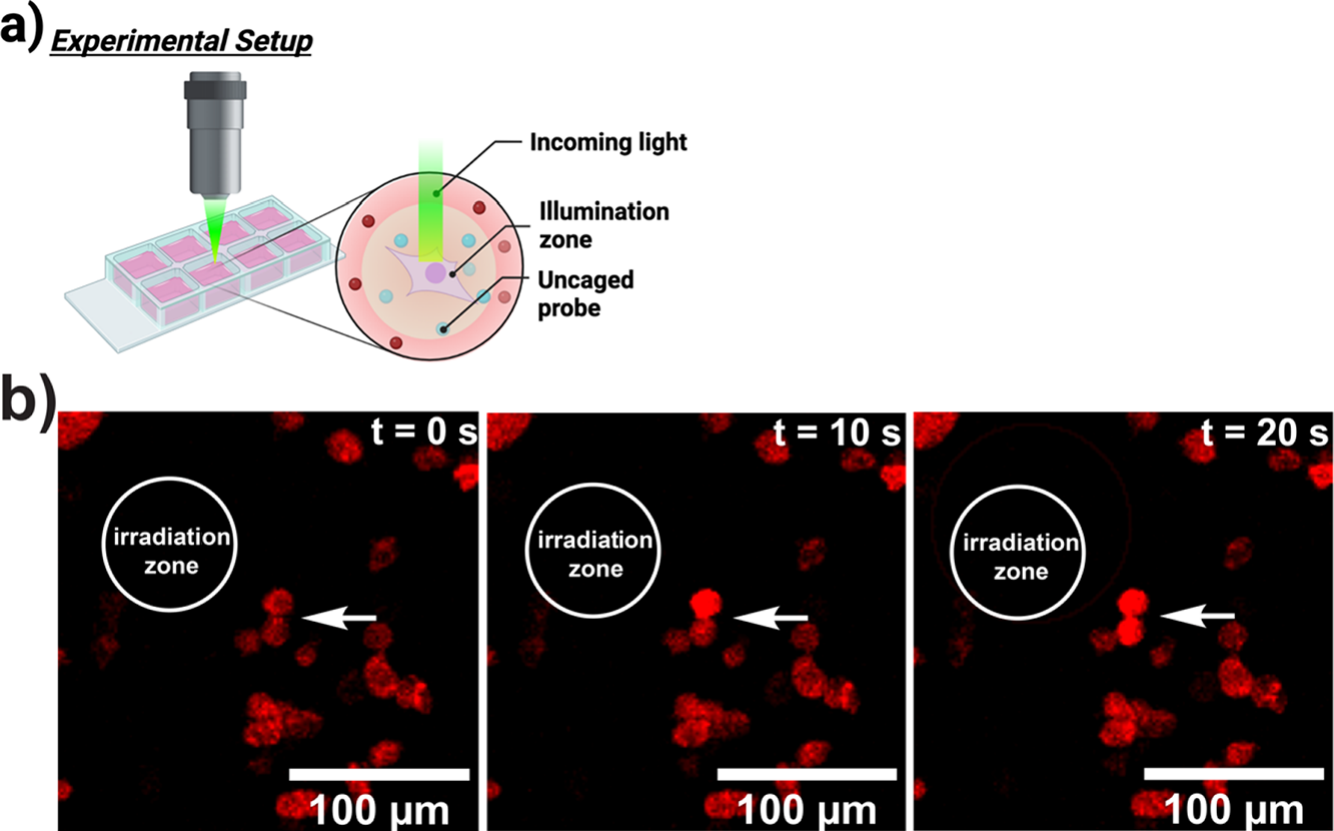
Live cell microscopy of 5-HT_2C_ activation. (a) Laser-scanning confocal microscopy enables discrete areas of uncaging. (b) Photoinduced release of WAY-161503 produces highly localized areas with WAY-161503 concentrations high enough to induce 5-HT_2C_ activation and subsequent intracellular calcium mobilization. White circles indicate areas of irradiation event, and white arrows point to cells demonstrating increased calcium flux.

We next determined the spatial resolution for cell activation with an uncaging event. Importantly, 5-HT_2C_ activation was only observed within an approximately 100 μm radius beyond the focus of irradiation (Figure 5a,b, quantitation in S4). This provides a high level of spatial control of activation and could serve as a powerful tool for studying cell–cell communication.

**Figure 6:**
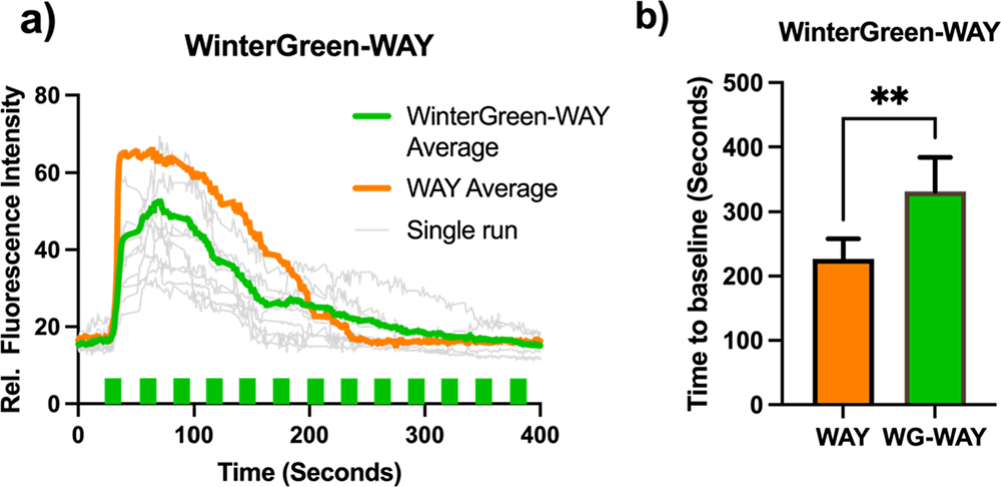
Long time-scale WinterGreen-WAY uncaging. (a) WinterGreen-WAY (**5**) uncaging induces lower maximum concentrations of intracellular calcium, but repeated irradiation can produce significantly longer calcium flux durations. Green blocks on line graph indicate events of 488 nm irradiation. 800 nM WAY-161503 injected into cell culture media as a control to determine calcium flux duration using traditional drug dosing techniques. (b) Bar chart of calcium flux durations comparing 800 nM WAY-161503 and WinterGreen-WAY (**5**). Average curves were generated as the average relative fluorescence intensity of 10 cells where basal fluorescence was normalized.

Repeated 200 ms irradiation in the presence of WinterGreen-WAY elicited extended calcium signaling, with a statistically significant longer calcium flux duration than 800 nM WAY-161503 (Figure 6). This indicated light-dose-dependent signaling for 5-HT_2C_ that could be achieved without deterioration of the response or high global drug concentration. Quantitation indicated the amount of drug released corresponds to approximately 300 nM of free drug (Figure S7). Overall, this demonstrates the utility of this tool as a method of performing experiments of long duration and repeatable dosing without the need to exchange cell culture media, an experimentally simple setup and mode of stimulus.

#### Photoactivation of WinterRed-NDMC **(12)**

**Figure 7:**
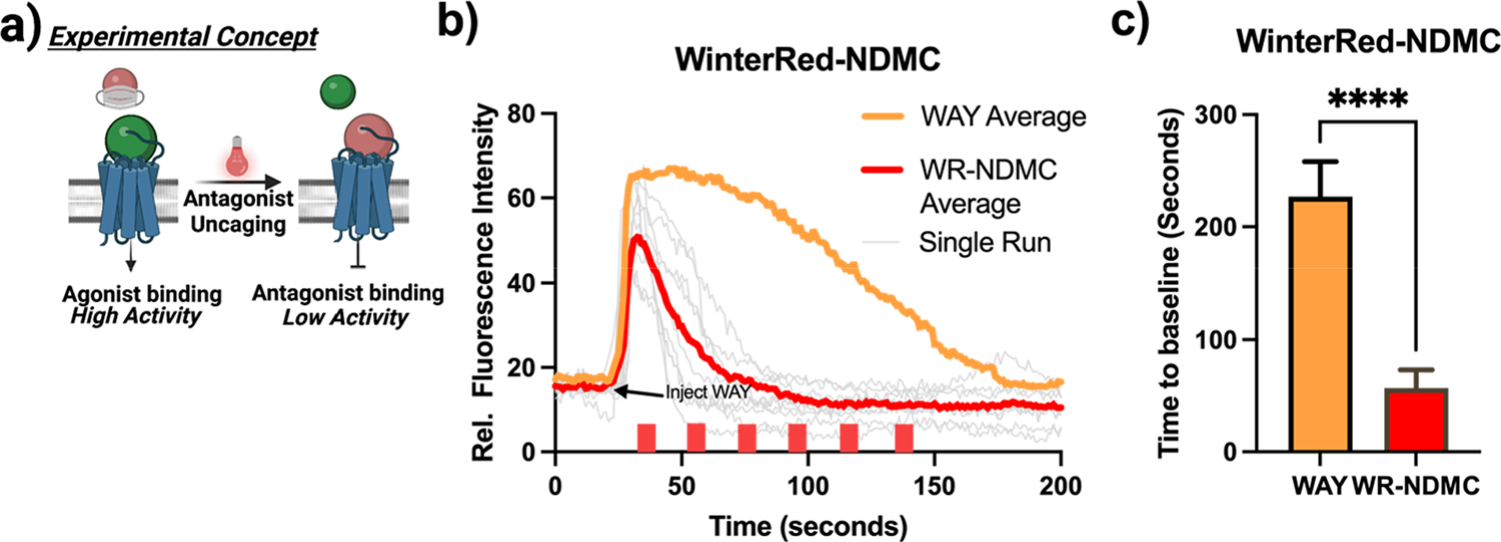
In vitro WinterRed-NDMC uncaging. (a) Schematic of experimental design: active antagonist is unveiled in the media, which will compete for active site binding. (b) Line graph comparing 800 nM WAY-161503 injection with (red line) and without (orange line) WinterRed-NDMC uncaging. Red blocks indicate events of 639 nm irradiation. (c) Bar chart comparing calcium flux durations of 800 nM WAY-161503 injection with and without WinterRed-NDMC uncaging. Average curves were generated as the average relative fluorescence intensity of 10 cells where basal fluorescence was normalized.

With the kinetics of WinterGreen-WAY uncaging defined, we sought to test WinterRed-NDMC uncaging under cell culture conditions. As an initial test of WinterRed-NDMC antagonistic efficacy, we designed a competition assay to test for spatially controlled NDMC binding of 5-HT_2C_ against WAY-161503. Media was loaded with 2.10 μM WinterRed-NDMC, 800 nM WAY-161503 was injected into the media, then the entire field of view was irradiated with 639 nm light to uncage NDMC. The concentration of WinterRed-NDMC corresponds to the concentration in the solution postfiltration based on a standardization curve (Figure S11). The calcium flux duration was compared to that of a sample containing no WinterRed-NDMC. A statistically significant decrease in both calcium flux duration and amplitude of calcium signaling was observed (Figure 7). From this, we concluded that the photolysis reaction of WinterRed-NDMC is active in cell culture media and demonstrated spatial modulation of calcium signaling within a large population of 5-HT_2C_-expressing cells.

#### Orthogonal Activation of WinterGreen-WAY and WinterRed-NDMC

**Figure 8:**
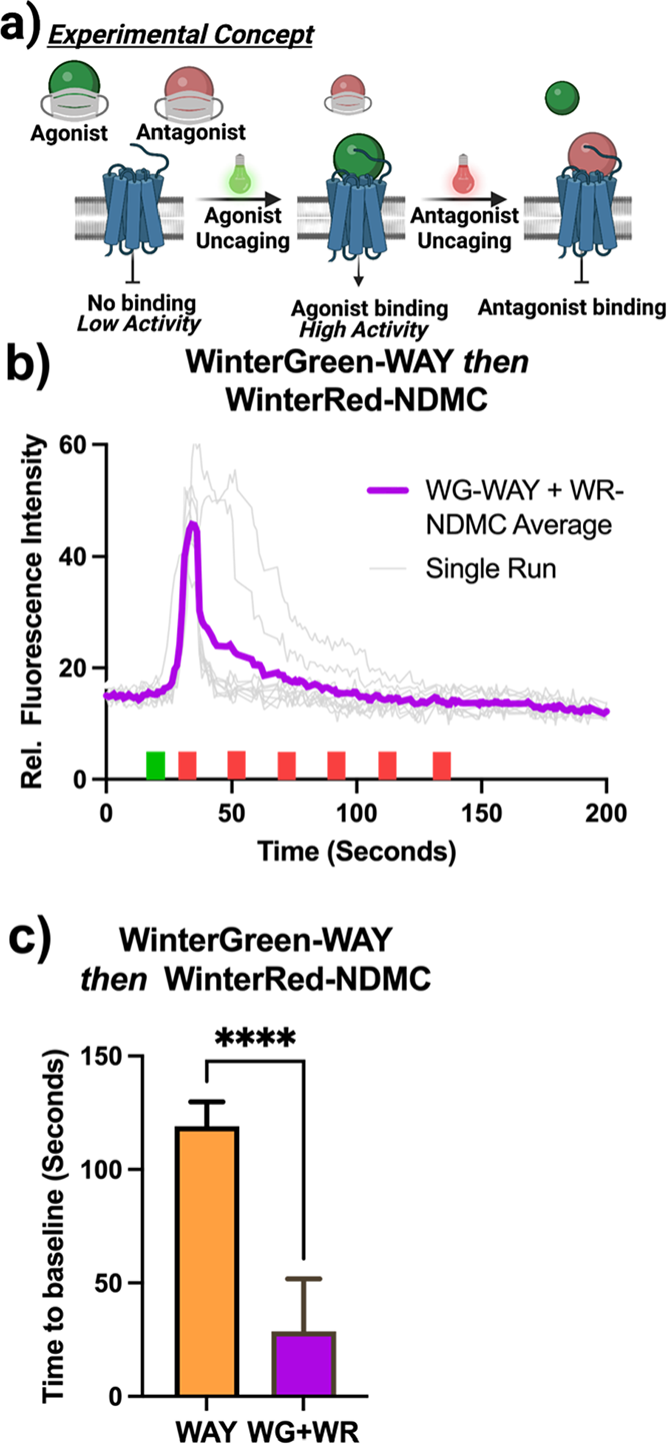
In vitro dual-wavelength uncaging. (a) Schematic of experimental design: active agonist is unveiled in the media to bind 5-HT_2C_, immediately followed by unveiled antagonist, which will compete for active site binding. (b) Line graph depicting dual-wavelength WinterGreen-WAY uncaging preceding WinterRed-NDMC uncaging. Green and red blocks indicate events of 488 nm and 639 nm irradiation, respectively. (c) Bar chart comparing calcium flux durations of 800 nM WAY-161503 injection with dual-wavelength WinterGreen-WAY and WinterRed-NDMC uncaging. “Average” curves were generated as the average relative fluorescence intensity of 10 cells where basal fluorescence was normalized.

With both compounds confirmed to be photoactive under cell culture conditions, we proceeded to investigate how they could be used in tandem to demonstrate a high degree of both spatial and temporal control of calcium signaling. To accomplish this, we loaded both WinterGreen-WAY and WinterRed-NDMC at a concentration of 3.71 and 2.10 μM, respectively, into cell culture media, chose a region of cells to activate by 488 nm uncaging, then immediately upon activation irradiated the same region with 639 nm light. The result of this is markedly tighter control of calcium flux durations and amplitude (Figure 8). Here, the on/off or activated/inactivated state of the receptor is controlled by pulses of light. This not only represents a novel avenue to reproducibly control the activation and signaling pattern of the 5-HT_2C_ receptor but also allows precise spatial control of the population induced.

## Conclusions

Photoactivatable forms of WAY-161503 and NDMC were designed as a system that allows for spectral multiplexing, enabling tight spatiotemporal control of 5-HT_2C_ calcium signaling. WinterGreen-WAY and WinterRed-NDMC were synthesized, characterized, and tested for loss of bioactivity at physiologically relevant concentrations. The kinetics of photolysis were characterized using HPLC and confirmation of functional drug release was performed in live cell culture. Both compounds had acceptable photolysis half-lives of 4.565 s for WinterGreen-WAY and 16.26 s for WinterRed-NDMC, and quantitatively photolyzed in under 25 and 75 s, respectively. Importantly, both compounds remained stable in solution for 48 h in the dark, and WinterGreen-WAY could release physiologically relevant payloads with a 200 ms pulse of 488 nm light in cell culture media. WinterGreen-WAY mediated the activation of 5-HT_2C_ signaling similar to that of commercially available WAY-161503, and with repeated 200 ms pulses of 488 nm light, demonstrated longer calcium flux durations than that of 800 nM WAY-161053. This indicated light-dose-dependent signaling for 5-HT_2C_ that could be achieved without deterioration of the response or high global drug concentration. Upon 639 nm irradiation, WinterRed-NDMC was demonstrated to diminish the effect of up to 800 nM WAY-161503 injected into cell culture media. Importantly, tandem uncaging of WinterGreen-WAY then WinterRed-NDMC produced markedly shorter calcium flux duration and amplitude, with precise spatial control. These tools enable a greater degree of spatiotemporal control of 5-HT_2C_ modulation and will allow for more detailed studies of receptors signaling, interactions with other proteins, and native physiology. Future studies will focus on applying this system to spatiotemporally control synaptic transmission of primary neurons on a receptor subtype-specific level.

## Methods

For general synthesis methods, see Supporting Information.

### *N*-Desmethylclozapine (9)

Compound **9** was prepared from **8** following the procedure of McRobb et al.^27^ The spectra matched those reported previously.

### WinterGreen-WAY (7)

To a solution of **5** (14.9 mg, 0.055 mmol, 1.0 eq) stirring in dry THF were added 4-dimethylaminopyridine (4.6 mg, 0.055 mmol, 1.0 eq) and **6** (20 mg, 0.055 mmol, 1.0 eq). The reaction was stirred at room temperature overnight. The reaction mixture was washed with water and extracted with ethyl acetate. The organic layer was dried over magnesium sulfate, and the solvent was removed under vacuum. The crude mixture was purified using silica gel column chromatography with 0–50% ethyl acetate in hexanes as the eluent to yield **7** as a bright red solid (0.0319 mmol, 58% yield). ^1^H NMR (400 MHz, CDCl_3_) 6.85 (s, 1H), 6.80 (m, 1H), 6.08 (s, 2H), 5.39 (s, 2H), 3.49 (m, 2H), 3.07 (t, 2H, *J* = 12.8 Hz), 2.63 (S, 1H), 2.47 (s, 6H), 2.38 (s, 6H), 2.18 (t, 2H, *J* = 2.2 Hz), 1.25 (s, 6H), 0.20 (s, 6H). ^13^C NMR (100 MHz, CDCl3) 153.3, 137.1, 134.8, 131.4, 127.3, 125.7, 123.6, 123.2, 122.7, 116.6, 113.9, 69.5, 56.4, 53.8, 44.4, 31.8, 29.7, 29.3, 25.4, 16.6, 15.9, 15.8, 14.1, 9.5. UV–visible λ_max_ = 515 nm. Fluorescence excitation/emission = 491 nm/529 nm. HPLC trace is shown in the Supporting Information on page S-16.

### WinterRed-NDMC (12)

To a solution of **9** (10 mg, 0.032 mmol, 1.0 eq) stirring in dry THF was added carbonyldiimidazole (7.7 mg, 0.048 mmol, 1.5 eq). The reaction was stirred under argon for 2 h. Then, 4-dimethylaminopyridine (3.8 mg, 0.032 mmol, 1.0 eq) and **11** (16.8 mg, 0.032 mmol, 1.0 eq) were added. The reaction was stirred at room temperature overnight. The reaction mixture was washed with water and extracted with ethyl acetate. The organic layer was dried over magnesium sulfate, and the solvent was removed under vacuum. The crude mixture was purified using silica gel column chromatography with 0–50% ethyl acetate in hexanes as the eluent to yield **1**2 as a dark green solid (0.017 mmol, 52% yield). ^1^H NMR (400 MHz, CDCl_3_) 7.48 (d, 4H, *J* = 8.6 Hz), 7.44 (s, 1H), 7.30 (d, 1H, *J* = 7.4), 7.24 (d, (1H, *J* = 8.0 Hz), 7.07 (m, 3H), 6.99 (s, 4H), 6.84 (dd, 1H, *J* = 10.6 Hz), 6.80 (d, 1H, *J* = 7.8 Hz), 6.73 (m, 7H), 6.60 (d, 1H, *J* = 8.4 Hz), 5.41 (s, 2H), 5.02 (s, 1H), 4.91 (m, 1H), 3.51 (m, 7H), 3.03 (s, 12H), 2.59 (s, 2H), 2.45 (s, 5H), 2.28 (s, 5H), 1.26 (s, 6H), 0.86 (m, 3H), 0.47 (s, 6H). ^13^C NMR (100 MHz, CDCl3) 155.1, 152.9, 151.5, 151.4, 150.8, 150.6, 150.6, 141.4, 140.4, 136.1, 135.8, 135.7, 133.4, 132.2, 130.0, 129.1, 128.4, 128.3, 128.2, 126.9, 125.6, 125.5, 123.5, 123.2, 120.1, 118.5, 117.1, 112.4, 67.9, 59.8, 40.4, 34.5, 30.2, 29.7, 25.6, 21.2, 16.1. UV–visible λ_max_ = 672 nm. Fluorescence excitation/emission = 683 nm/730 nm. HPLC trace is shown on page S-16.

#### HPLC Photolysis Characterization

For WinterGreen-WAY characterization, the compound was taken up in DMSO as a 1.5 mg/mL solution and sterile-filtered to remove any aggregates. 100 μL aliquots were irradiated with a 4500 mW/cm^2^ spot Hg-arc light source (Hamamatsu LC8) for various time points. Aliquots were then purified on a Waters HPLC, with a Waters 2545 binary gradient pump equipped with Waters 2489 UVVis Detector. The column was a Phenomenex C18 column (4.6 × 50 mm) run with a gradient of 5–95% acetonitrile in water for 20 min. Percent WinterGreen-WAY remaining was calculated from the integrated peak area.

For WinterRed-NDMC characterization, compound was taken up in ethyl acetate as a 1.5 mg/mL solution and sterile-filtered to remove any aggregates. Irradiation procedure and HPLC instrument were the same as above. The column was a Phenomenex Luna 5 μm silica column (4.6 × 50 mm) run with a gradient of 5–95% ethyl acetate in hexanes for 20 min. Percent WinterRed-NDMC remaining was calculated from the integrated peak area.

#### TANGO Assay Biochemical Characterization

HTLA cells were a gift from the laboratory of G. Barnea and were maintained in DMEM supplemented with 10% fetal bovine serum (FBS), 100 U/mL penicillin and 100 μg/mL streptomycin, 2 μg/mL puromycin, 100 μg/mL hygromycin B, and 100 μg/mL G418, in a humidified atmosphere at 37 °C in 5% CO^2^. On day 1, cells were plated at a density of 1x10^5^ cells/cm^2^ in a black wall, clear bottom 96 well plate (Nunc). On the following day (day 2), cells were transfected with a 10× solution of 3:1 mixture of HTR2C-TANGO (Addgene #66411):Optifect Transfection Reagent (Thermo) in un-supplemented DMEM. On day 3, 1× drug stimulation solutions were prepared in filter-sterilized unupplemented DMEM. The transfection media was shaken or aspirated from the wells, and drug stimulation solutions were gently added. On day 4, drug solutions were removed from one well every 10 s (to maintain consistency of incubation time) and 50 μL per well of Bright-Glo solution (Promega) diluted 20-fold in HBSS was added. After incubation for 2 min at room temperature, luminescence was counted with an integration time of 10 s in a Spectramax i3× plate reader (Molecular Devices). Drug concentrations were experimentally measured in triplicate. Statistical analysis was performed using GraphPad Prism 9.

#### Uncaging in Cell Culture

HEK293T cells were maintained in DMEM supplemented with 10% FBS, 100 U/mL penicillin and 100 μg/mL streptomycin, in a humidified atmosphere at 37 °C in 5% CO2. On day 1, cells were plated at a density of 4 × 10^4^ cells/cm2 in a poly-d-lysine-coated 18-well chambered coverslip (Ibidi). On the following day (day 2), cells were transfected with a 10× solution of 3:1 mixture of FRT-5HT-GFP (Addgene #79677):Optifect Transfection Reagent (Thermo) and a 3:1 mixture of jRCaMP-1a (Addgene # 61562):Optifect Transfection Reagent (Thermo) in unsupplemented DMEM. On day 3, the transfection media was removed and freshly prepared unsupplemented media loaded with either 3.7 μM WinterGreen-WAY, 2.1 μM WinterRed-NDMC, or a saturated solution in 90:10 DMEM:DMSO of both compounds was added to the wells. We note that this concentration of DMSO was necessitated by the poor solubility profile of the parent Bodipy dyes. This concentration of DMSO has previously been shown not to affect the viability of transfected HEK293T cells when exposed for 10 min or less, below our imaging times.^33^ Live cell uncaging and imaging were performed on a Zeiss LSM 980 with Airyscan 2. Basal fluorescence was recorded for 20 s followed by spot bleaching of the region of interest at 60% laser intensity using the appropriate wavelength. Nominal power for the 488 nm and 639 nm lasers used were 10.0 and 7.5 mW, respectively. Calcium response was recorded for 800 s after initial irradiation. Data extraction was completed with ImageJ. “Average” curves were generated from the average relative fluorescence intensity of 10 cells, where basal fluorescence was normalized.

## Supporting Information Available

General chemistry procedures, NMR spectra, HPLC spectra, UV–vis spectra, fluorescence spectra, quantification of percent cells activated, UV–vis concentration curve in cell culture media, plot showing multiple irradiation can evoke repeated calcium flux, recapitulation of the WinterRed-NDMC calcium flux curve, recapitulation of the WinterGreen-WAY calcium flux curve, alternate wavelength (spectral multiplexing) control, WinterGreen-WAY antagonist TANGO assay, 5-HT_2C_ unexpressed negative control, and serotonin positive controls in calcium and TANGO assay (PDF)
