# Supplementary material for: Photoactivatable Agonist–Antagonist Pair as a Tool for Precise Spatiotemporal Control of Serotonin Receptor 2C Signaling
Source: ACS Chem Neurosci. 2023 Sep 18;14(19):3665–73. doi: 10.1021/acschemneuro.3c00290 (PMC10557072; doi:10.1021/acschemneuro.3c00290)
Supplement: Supplementary file 1 — cn3c00290_si_001.pdf [file cn3c00290_si_001.pdf]

**Supporting Information**

# Photoactivatable Agonist-Antagonist Pair as a Tool for Precise Spatiotemporal Control of Serotonin Receptor 2C (5HT2C) Signaling

Spencer T. Kim[1], Emma J. Doukmak[1], Michelle Shanguhyia[1], Dylan J. Gray[1], Rachel C. Steinhardt[1*]

[1]Syracuse University, Syracuse, NY 13244
[*]To whom correspondence should be addressed, rcsteinh@syr.edu

**Table of Contents**


| | |
|---|---|
| Synthetic chemistry general experimental procedures | S-3 |
| UV-Vis and fluorescence spectra figs **S1-S3** | S-4 |
| Quantification of percent cells activated fig **S4** | S-7 |
| Multiple irraditations can evoke repeated calcium flux fig **S5** | S-8 |
| Recapitulation of WinterRed-NDMC calcium flux curve fig **S6** | S-9 |
| Recapitulation of WinterGreen-WAY calcium flux curve fig **S7** | S-10 |
| Serotonin positive controls in Calcium and TANGO assay fig **S8** | S-11 |
| 5-$HT_{2C}$ unexpressed negative control data fig **S9** | S-12 |
| WinterGreen-WAY antagonist TANGO assay **S10** | S-13 |
| Standard UV/Vis concentration curve in cell culture media **S11** | S-14 |
| Alternate wavelength (spectral multiplexing) control **fig S12** | S-15 |
| HPLC traces of WinterGreen WAY and WinterRed NDMC | S-16 |
| NMR Spectra | S-17 |

**Supporting information**

**Synthetic Chemistry General experimental procedures**

All reactions were performed in flame- or oven-dried glassware under positive pressure of nitrogen or argon unless otherwise noted. Dichloromethane, 1,2-dichloroethane, dimethylacetamide, *N,N,* dimethylformamide, triethylamine, and toluene were dried by columns packed with activated alumina on a solvent purification system. Anhydrous DMSO was purchased from Acros Organics in AcroSeal bottles. All reagents were used as purchased without further purification. Thin layer chromatography (TLC) was performed on Merck 60 F254 pre-coated silica gel plates, and plates were visualized with UV light and ninhydrin stain when appropriate. Flash-column chromatography was performed using silica gel (60 Å, 230-240 mesh, Merck KGA). HPLC runs were conducted on a Waters 2545 binary gradient pump equipped with UVVis Detector. Analytical runs were performed using a Phenomenex C18 column (4.6 x 50 mm) and a Phenomenex Luna 5μm silica column (4.6 x 50 mm). Separations were monitored at 325 nm and 254 nm. NMR spectra were recorded with Bruker Avance spectrometers using deuterated solvents. $^{1}H$ NMR spectra were recorded at 400 MHz as indicated. $^{13}C$ spectra were recorded at 100 MHz. $^{1}H$ NMR data are reported in the following order: chemical shift (δ ppm), multiplicity, coupling constant (Hz), and integration. $^{13}C$ NMR data are reported in terms of chemical shift. UV-Visible spectroscopy was preformed using an Agilent Technologies Cary 60 UV-Visible Spectrophotometer. Fluorescence spectroscopy was performed using a Horiba Scientific FluoroMax+ Spectrofluorimeter. The abbreviations used can be found in the document *JOC Standard Abbreviations and Acronyms*.

**Figure S1.** UV-Visible Spectra of WinterGreen-WAY (**a**) and WinterRed-NDMC (**b**)

**a)**

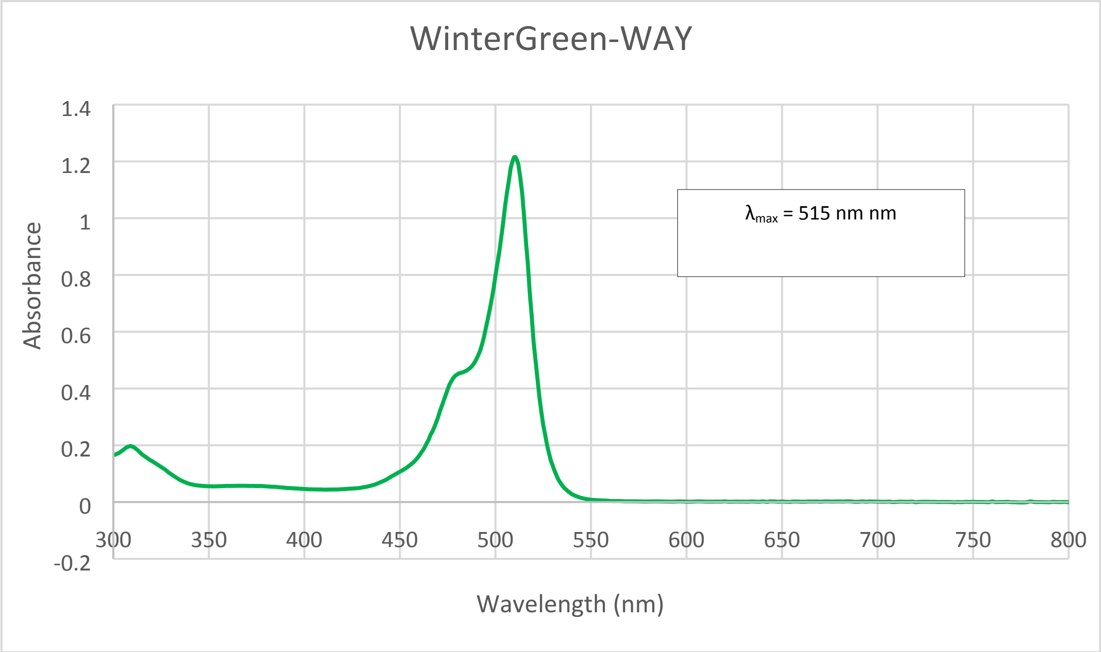


**b)**

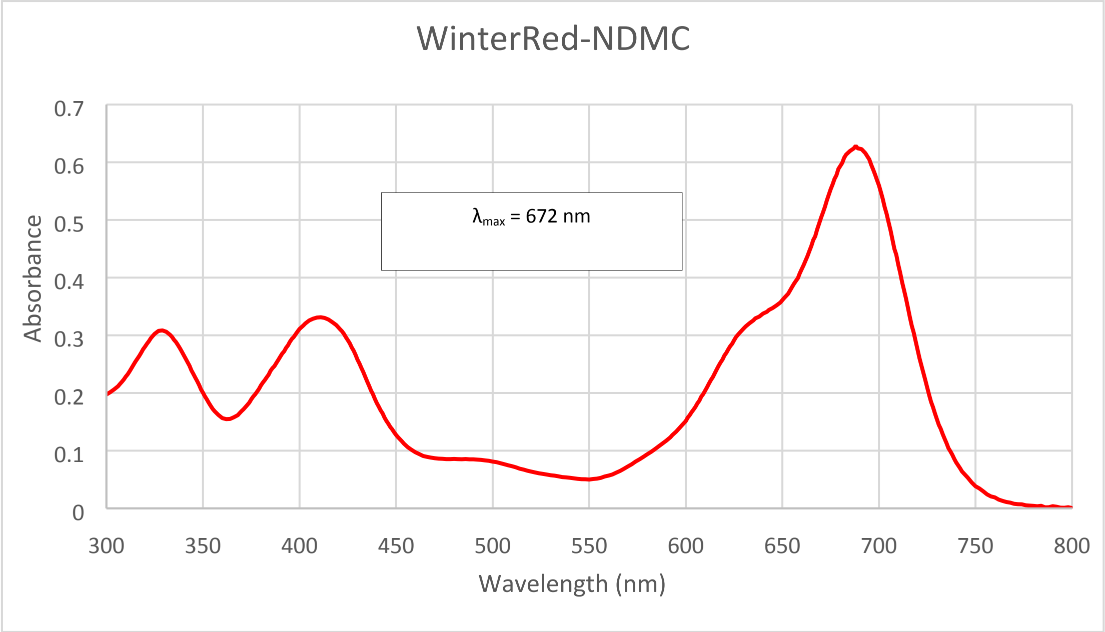

**Figure S2.** Fluorescence Spectra of WinterGreen-WAY (**a**) and WinterRed-NDMC (**b**)

**a)**

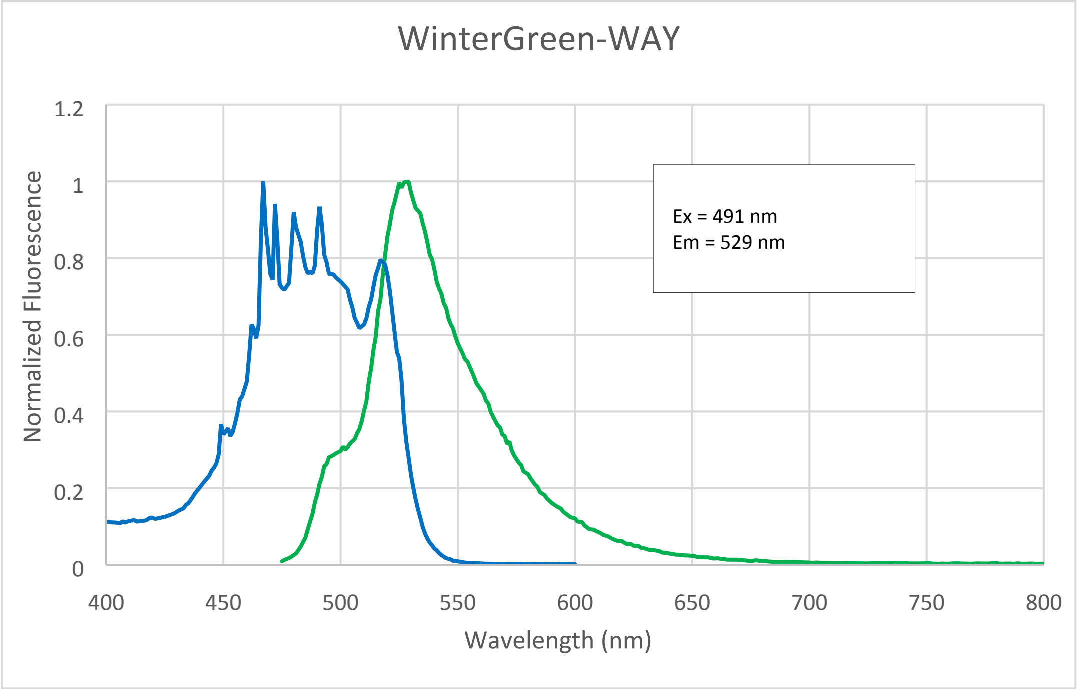


**b)**

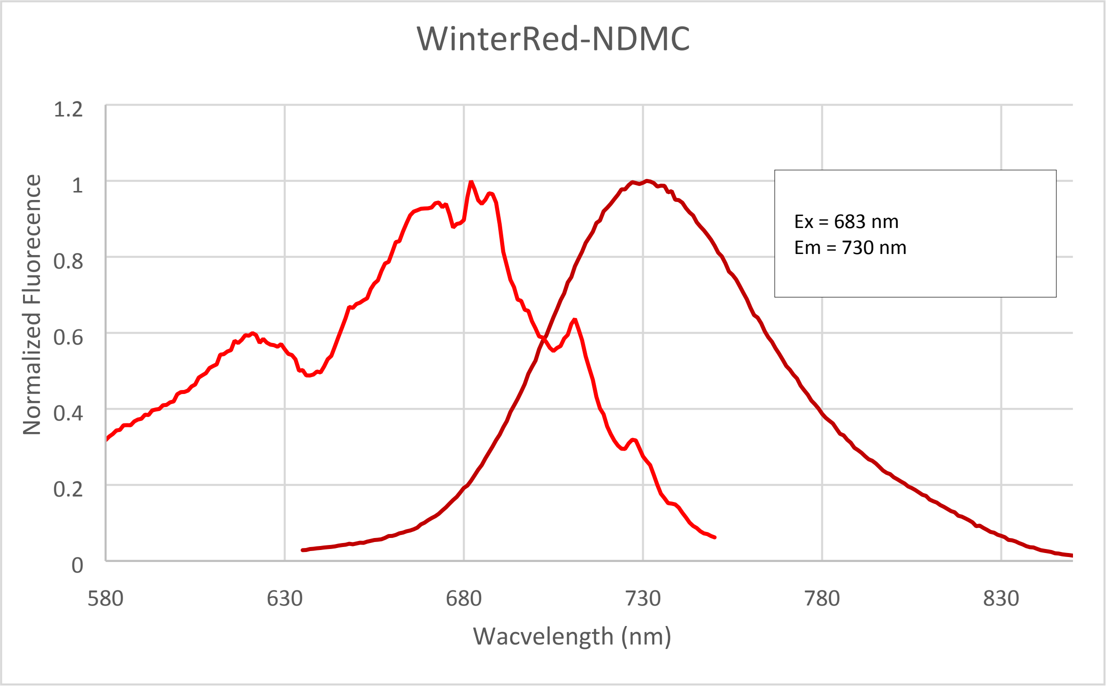

**Figure S3.** Combined UV-Visible and Fluorescence Spectra of WinterGreen-WAY (**a**) and WinterRed-NDMC (**b**)

**a)**

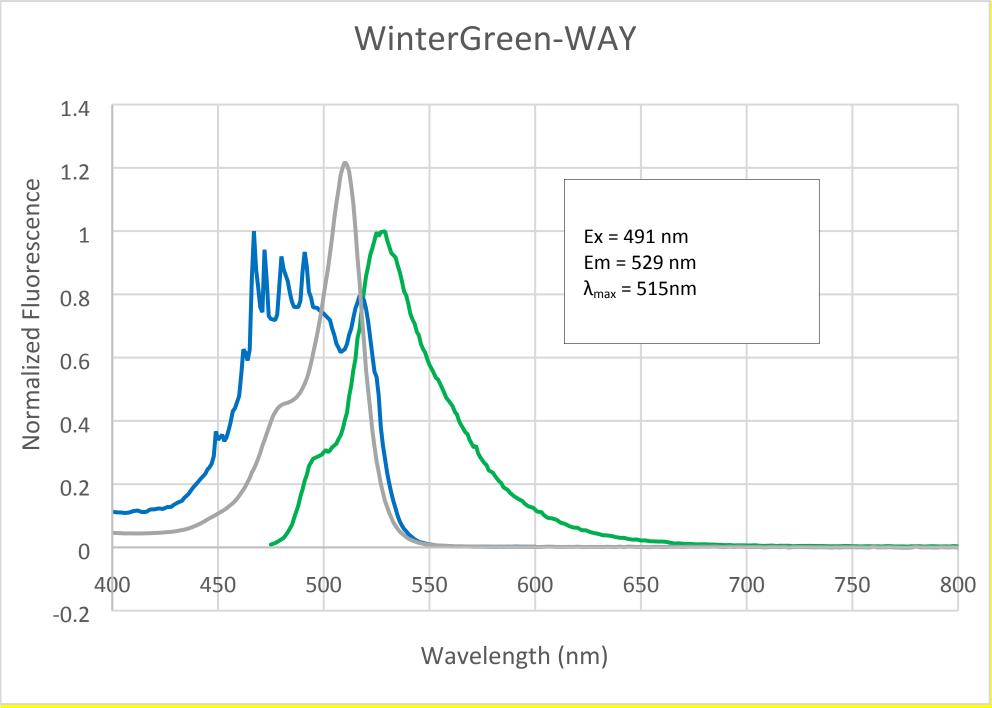


**b)**

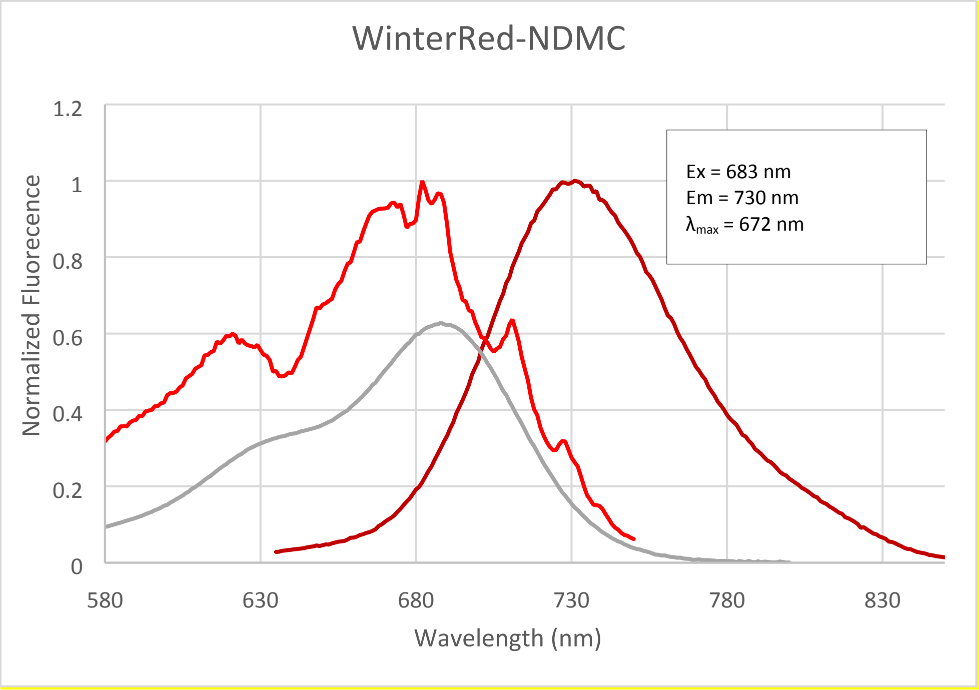

**Figure S4.** Quantification of percent of cells activated surrounding a region of irradiation in response to a 200 ms pulse of 488 nm light. To quantitate, we look at the number of cells activated in radii of different lengths from the focal point of the laser. To maximize cell viability and minimize the chance for experimental artefacts, the laser is never pointed directly at a cell. All cells within the radius are counted.

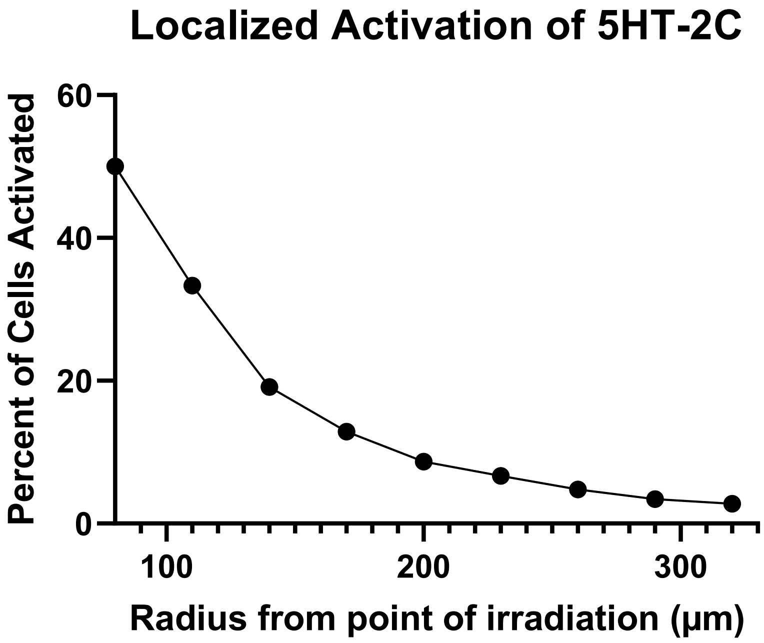

**Figure S5.** Repeated irradiation of WinterGreen-WAY is able to envoke subsequent events of calcium mobilization. Each colored line corresponds to an individual cell, in an ensemble of 10 cells.

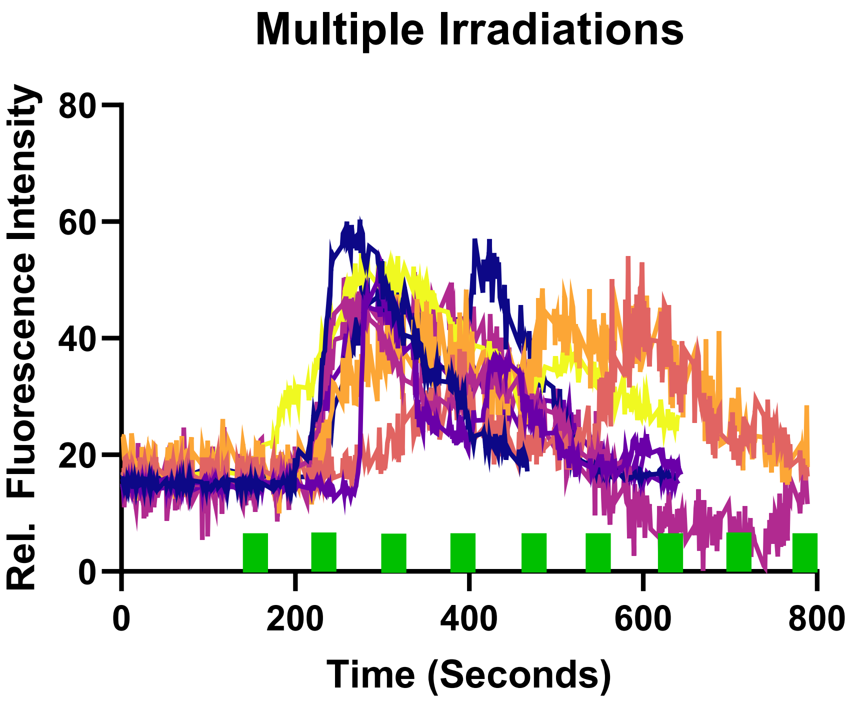

**Figure S6.** Recapitulation of parent WAY-161503/WinterRed-NDMC calcium flux curve through preincubation of 250 nM NDMC then 800 nM parent WAY-161503 addition.

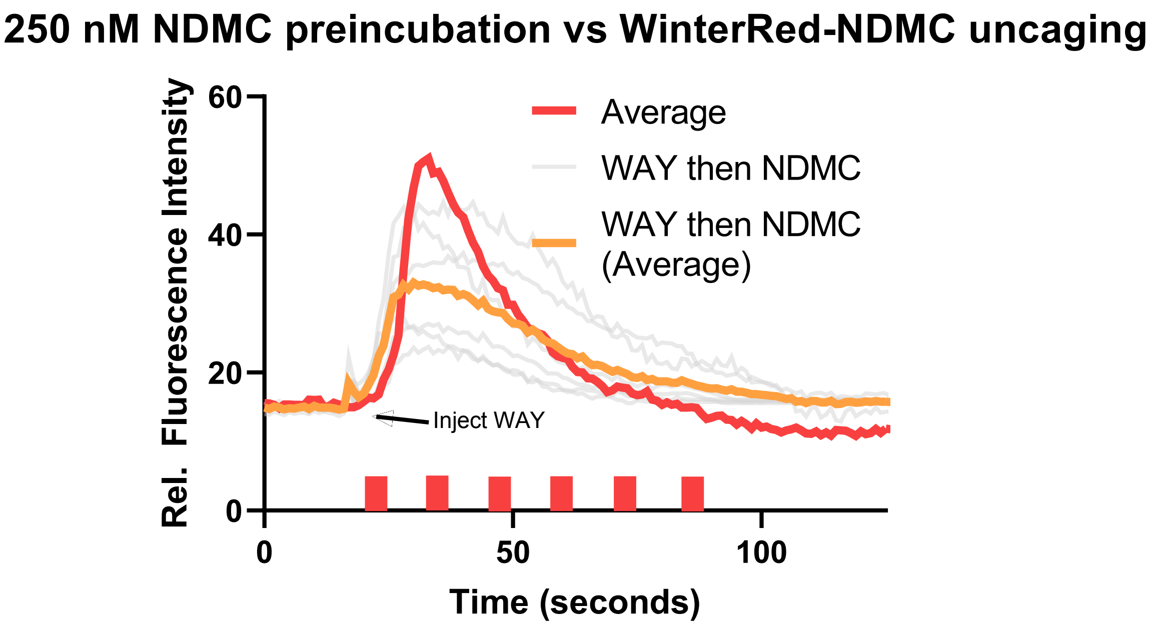

**Figure S7.** Recapitulation of 200 ms irradiation WinterGreen-WAY calcium flux curve through the addition of 300 nM parent WAY-161503

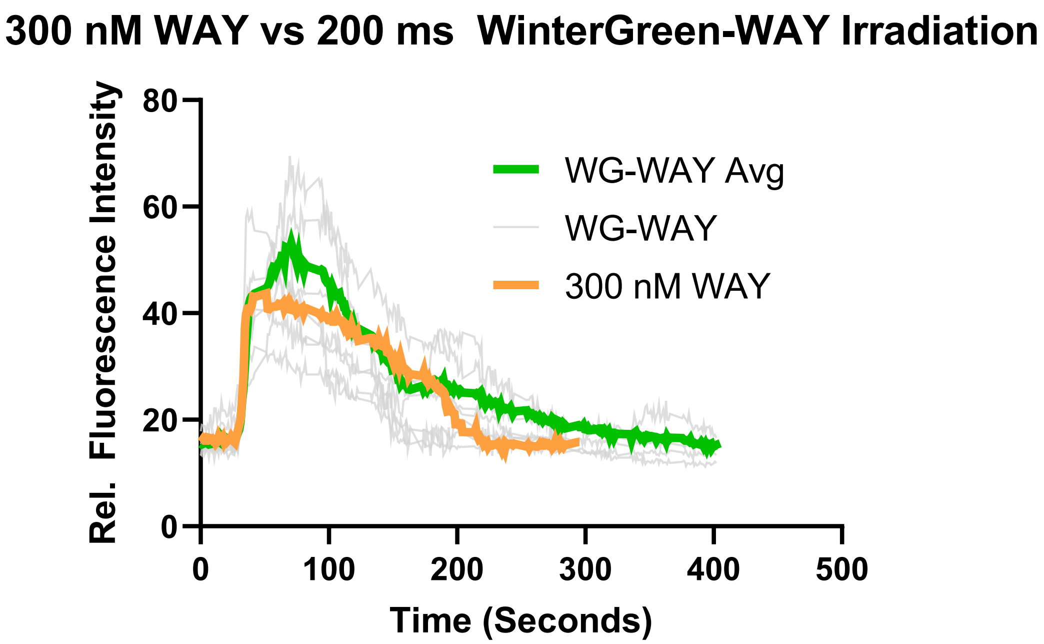

**Figure S8.** Serotonin (5-HT) positive control data for TANGO bioluminescence (**a**) and calcium flux microscopy (**b**).

**a)**

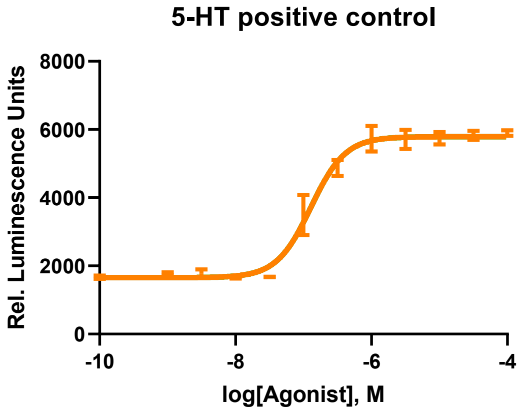


**b)**

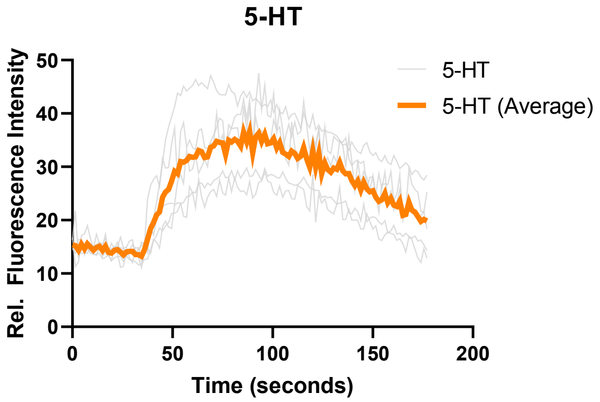

**Figure S9**. Negative control data against cells not expressing 5-HT$_{2C}$. TANGO bioluminescence (**a**) and calcium flux microscopy (**b-d**).

a)

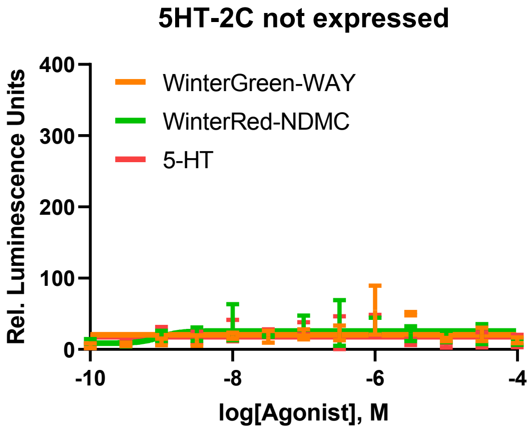


b)

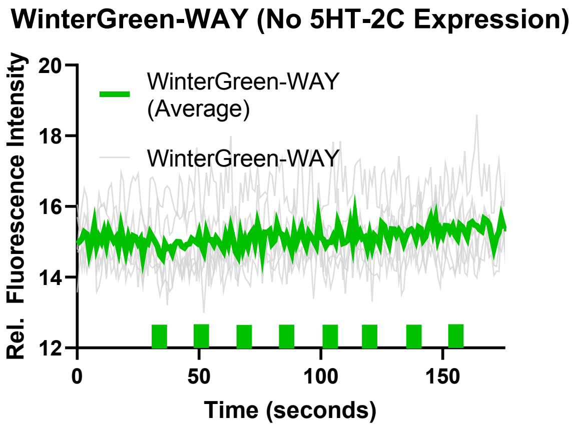


c)

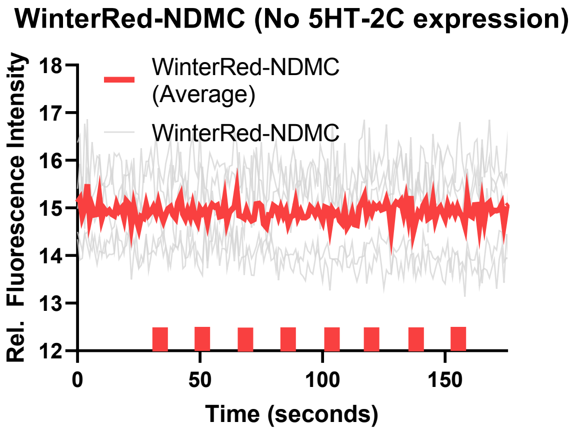

**Figure S9** continued.

**d)**

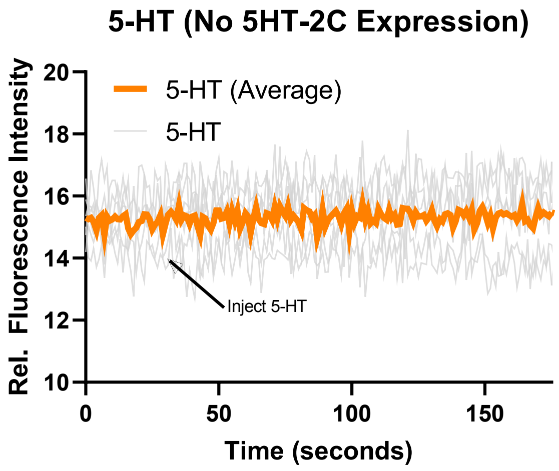


**Figure S10.** WinterGreen-WAY tested as an antagonist against WAY-161503 in the TANGO bioluminescence assay.

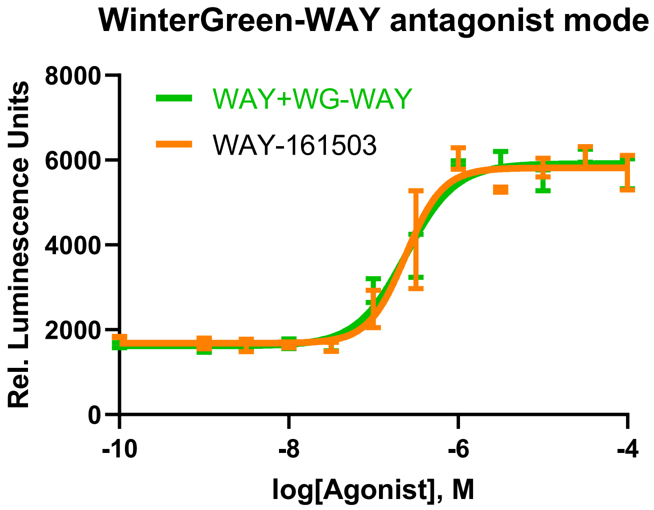

**Figure S11.** UV-Vis standard curve of WinterGreen-WAY (**a**) and WinterRed-NDMC (**b**) in DMEM containing 10% (v/v) DMSO. Solubility at room temperature was determined to be 3.71 µM and 2.10 µM, respectively.

**a)**

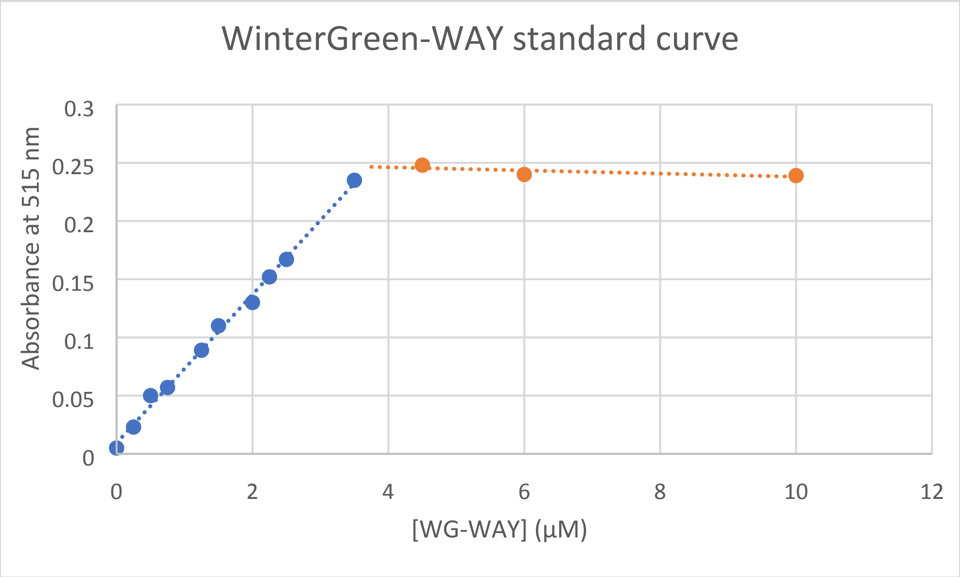


**b)**

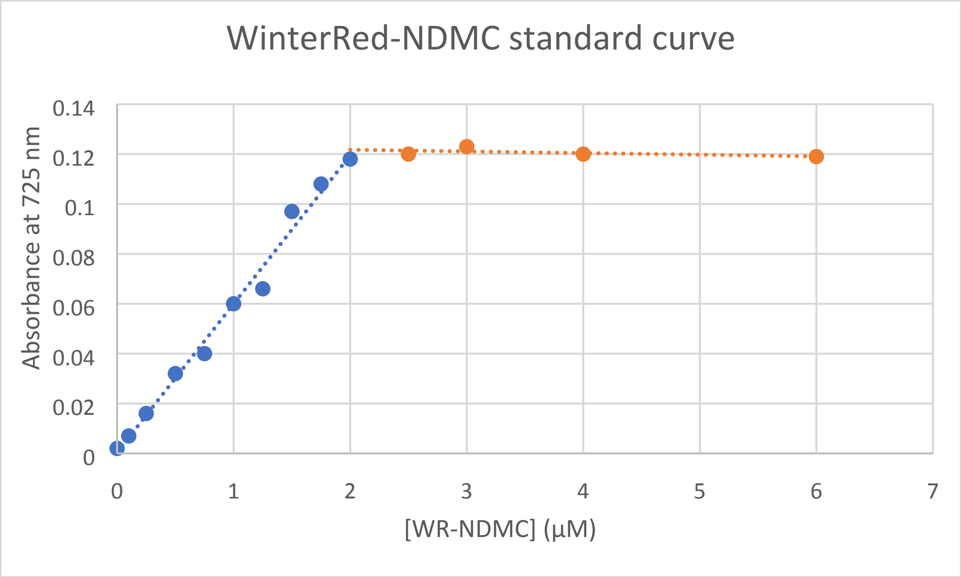

**Figure S12.** Uncaging control. The alternate laser wavelength does not uncage the corresponding compound.

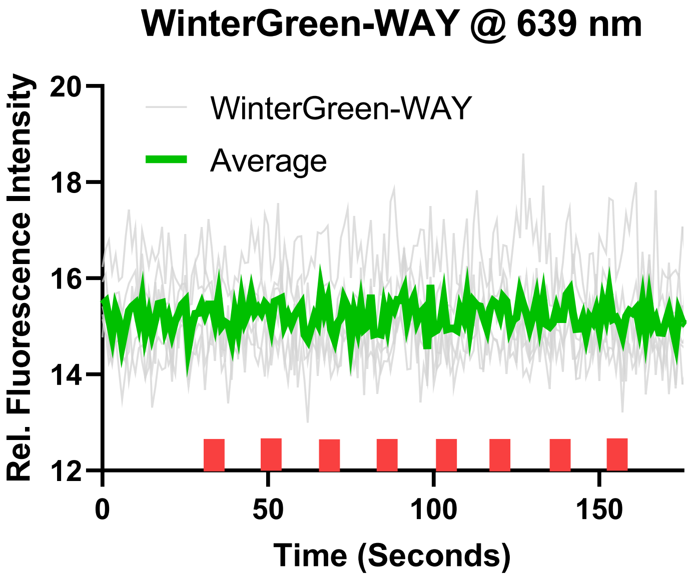


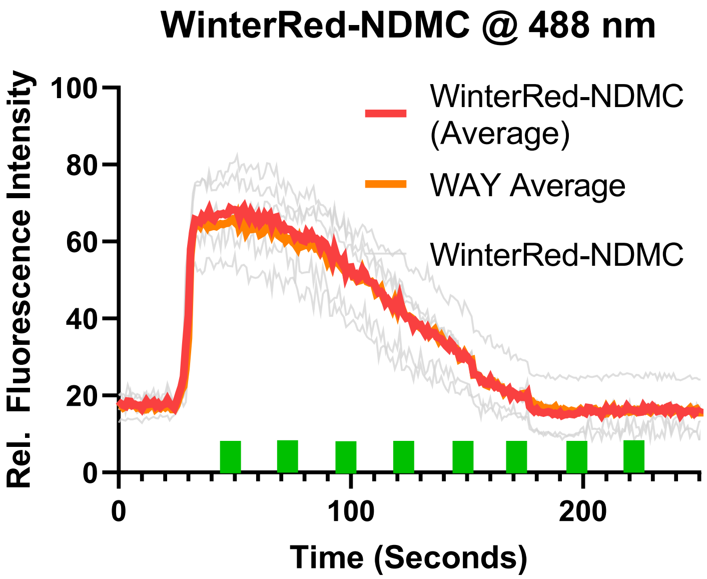

## HPLC trace of WinterGreen-WAY

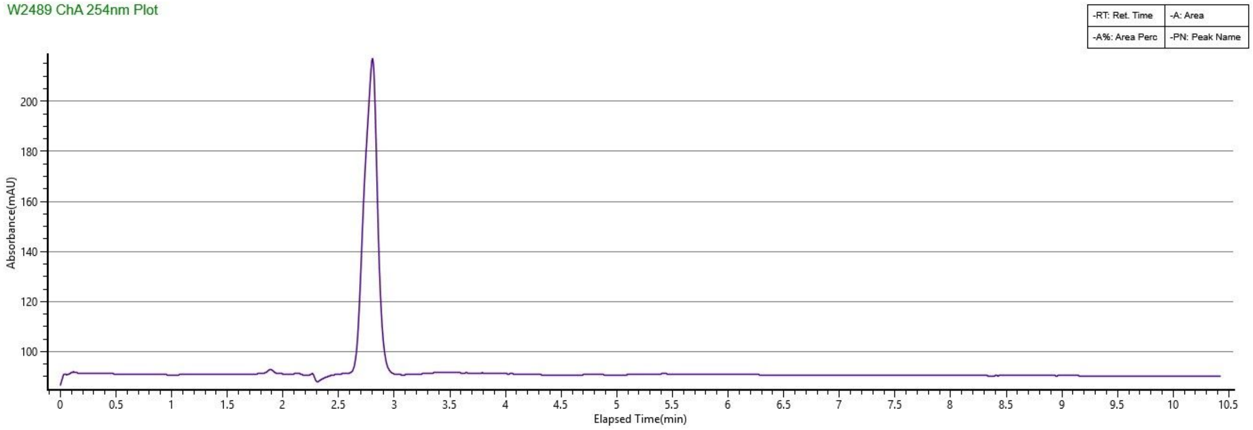


## HPLC trace of WinterRed-NDMC

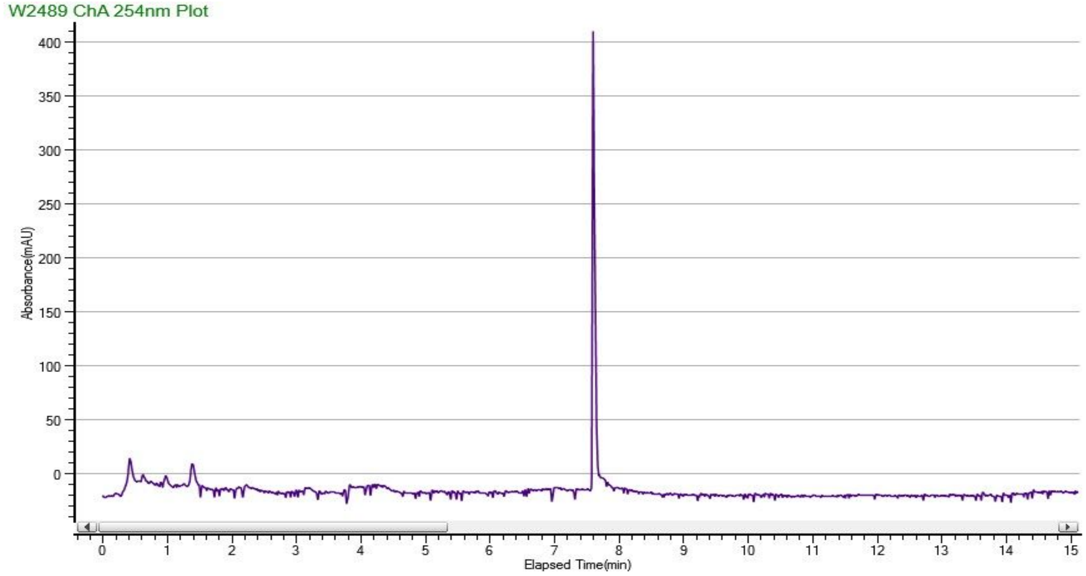

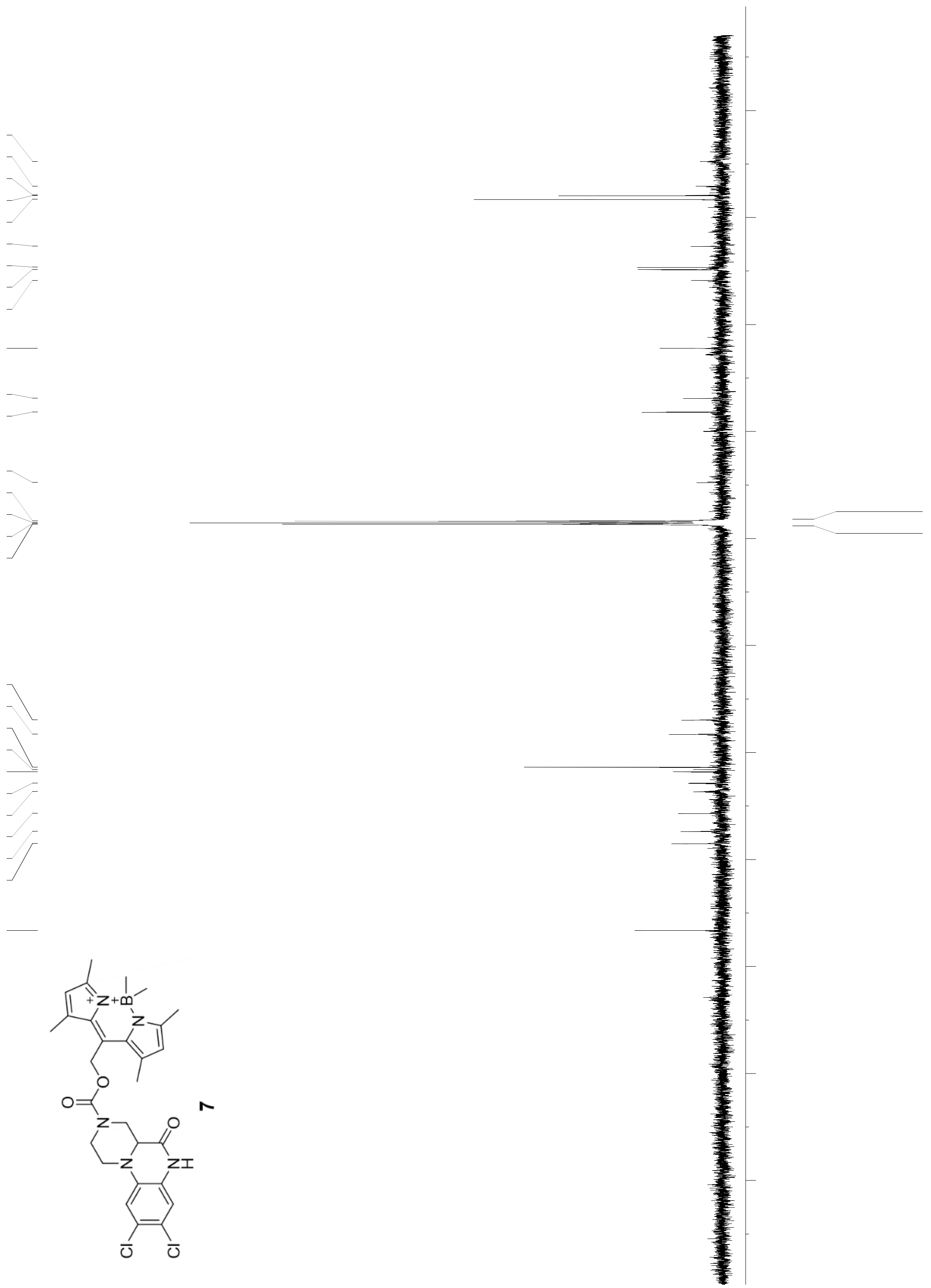

7

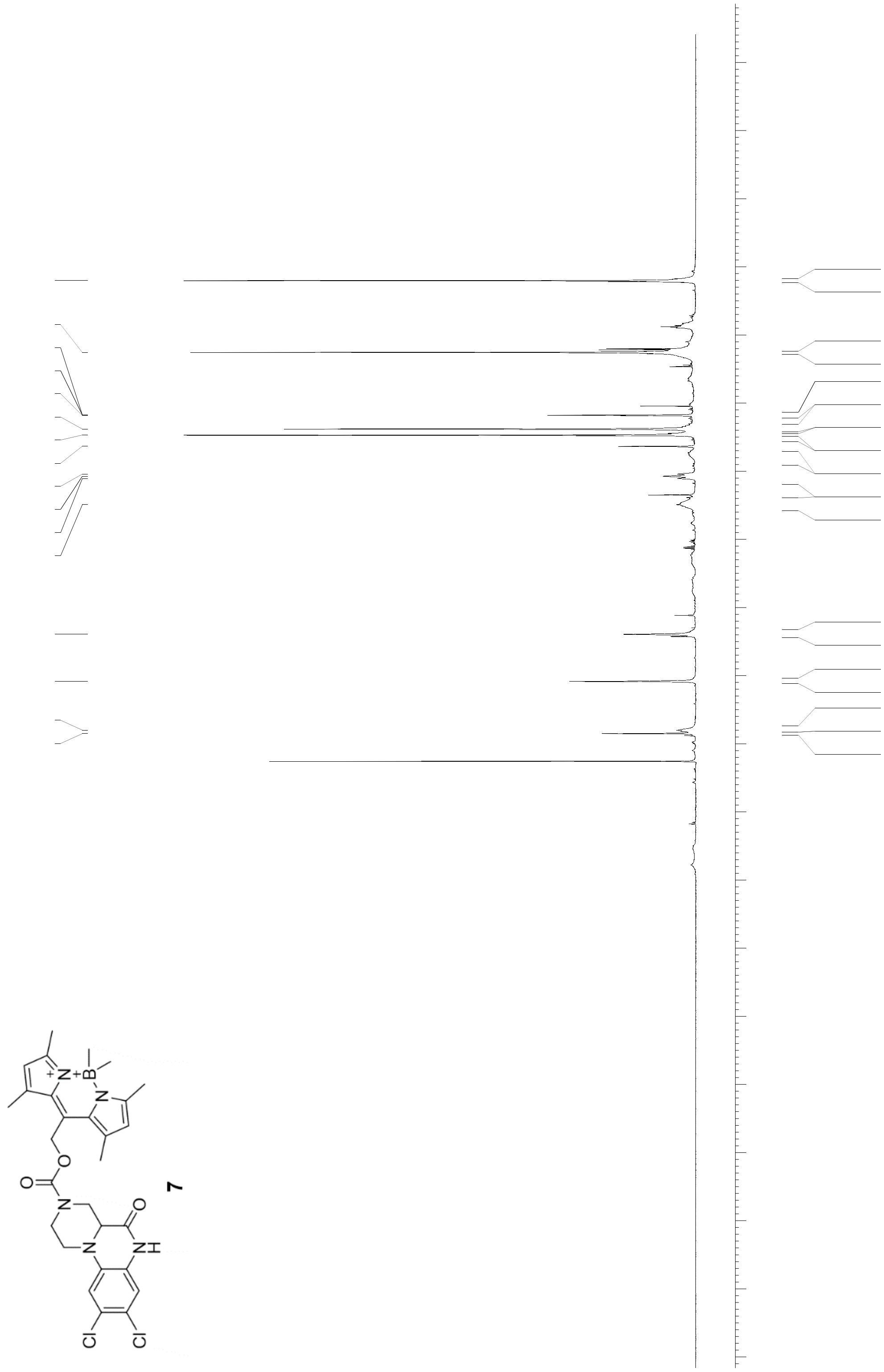

7

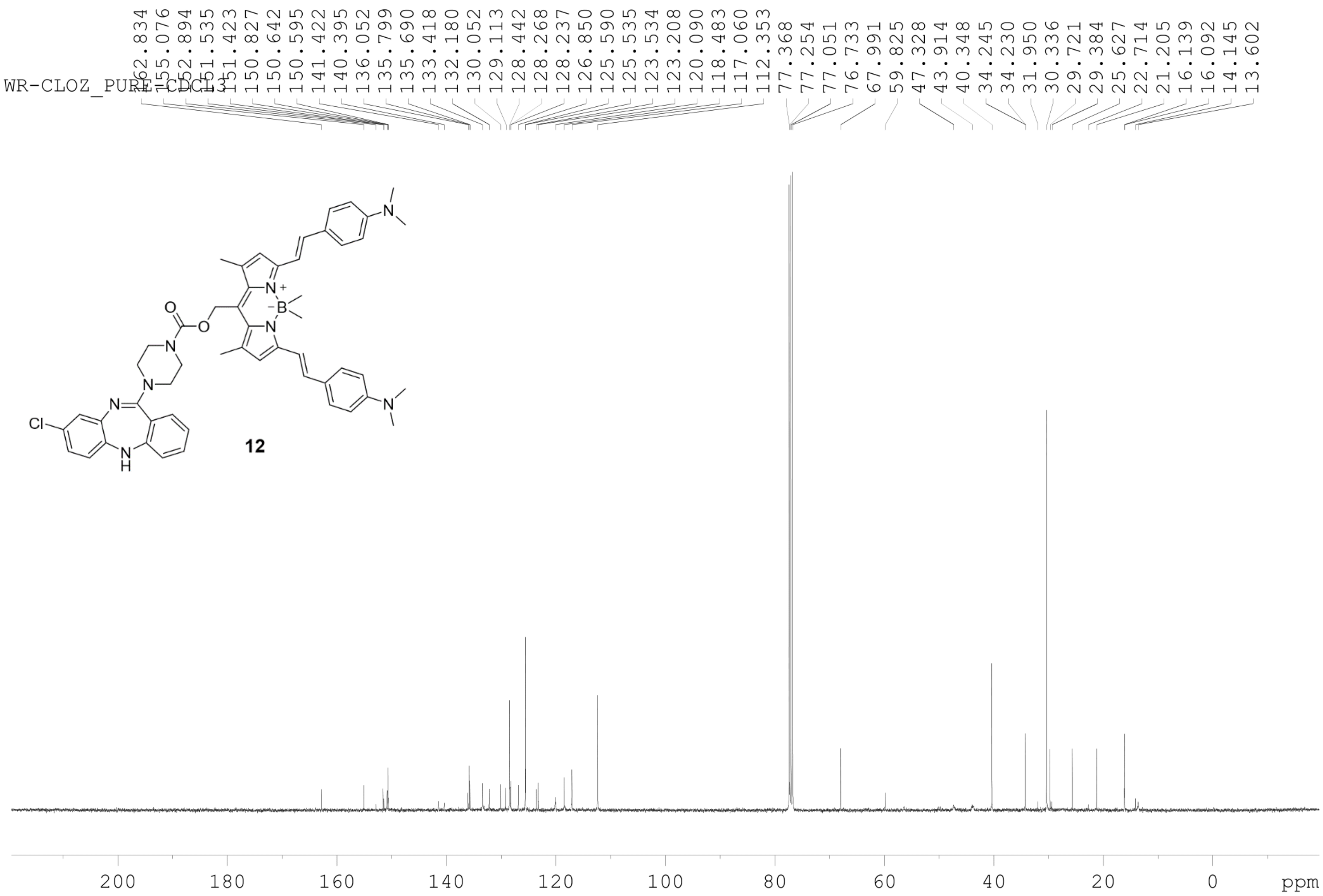
WR-CLOZ_PURE-CDCL3
162.834
155.076
152.894
151.535
151.423
150.827
150.642
150.595
141.422
140.395
136.052
135.799
135.690
133.418
132.180
130.052
129.113
128.442
128.268
128.237
126.850
125.590
125.535
123.534
123.208
120.090
118.483
117.060
112.353
77.368
77.254
77.051
76.733
67.991
59.825
47.328
43.914
40.348
34.245
34.230
31.950
30.336
29.721
29.384
25.627
22.714
21.205
16.139
16.092
14.145
13.602
12
200
180
160
140
120
100
80
60
40
20
0
ppm

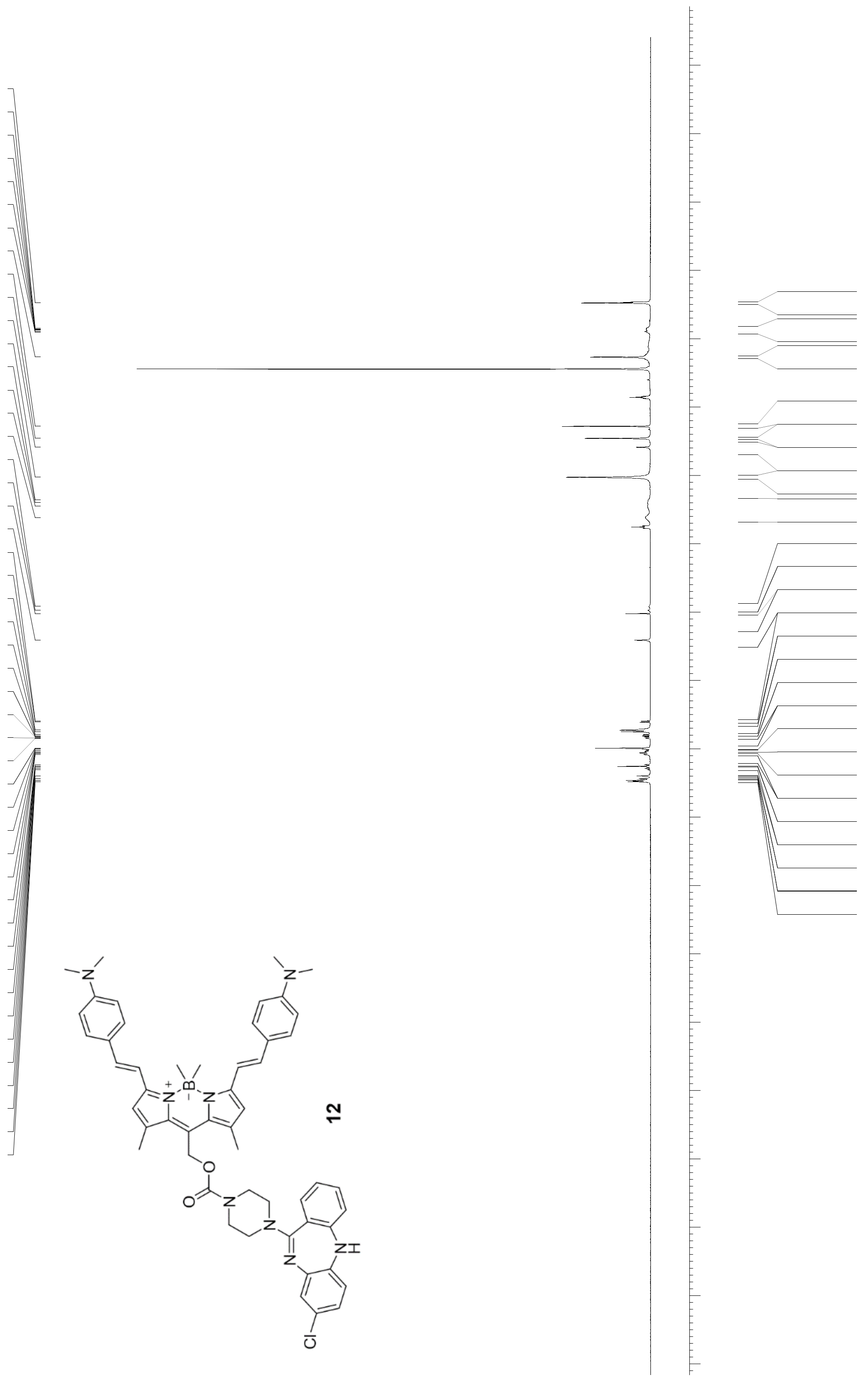
12
